# Examining Hemin and its Derivatives: Induction of Heme-Oxygenase-1 Activity and Oxidative Stress in Breast Cancer Cells through Collaborative Experimental Analysis and Molecular Dynamics Simulations

**DOI:** 10.1021/acs.jmedchem.4c00989

**Published:** 2024-08-19

**Authors:** Amir M. Alsharabasy, Panagiotis I. Lagarias, Konstantinos D. Papavasileiou, Antreas Afantitis, Pau Farràs, Sharon Glynn, Abhay Pandit

**Affiliations:** † CÚRAM, SFI Research Centre for Medical Devices, University of Galway, Galway H91 W2TY, Ireland; ‡ Department of ChemoInformatics, Novamechanics Ltd., Nicosia 1070, Cyprus; § Department of Chemoinformatics, Novamechanics MIKE, Piraeus 18545, Greece; ∥ Division of Data Driven Innovation, Entelos Institute, Larnaca 6059, Cyprus; ⊥ School of Biological and Chemical Sciences, Ryan Institute, University of Galway, Galway H91 TK33, Ireland; # Discipline of Pathology, Lambe Institute for Translational Research, School of Medicine, University of Galway, Galway H91 YR71, Ireland

## Abstract

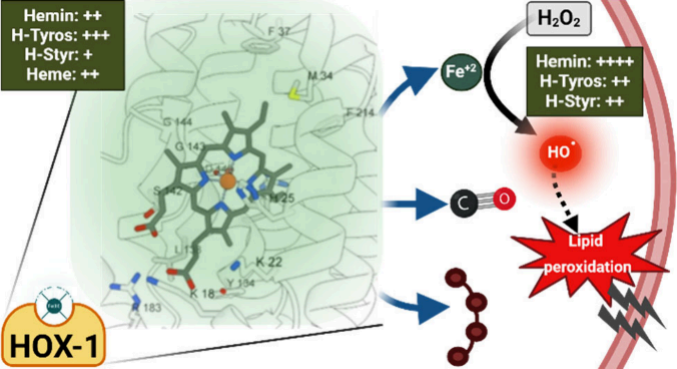

Hemin triggers intracellular reactive oxygen species (ROS) accumulation and enhances heme oxygenase-1 (HOX-1) activity, indicating its potential as an anticancer agent, though precise control of its intracellular levels is crucial. The study explores the impact of hemin and its derivatives, hemin-tyrosine, and hemin-styrene (H-Styr) conjugates on migration, HOX-1 expression, specific apoptosis markers, mitochondrial functions, and ROS generation in breast cancer cells. Molecular docking and dynamics simulations were used to understand the interactions among HOX-1, heme, and the compounds. Hemin outperforms its derivatives in inducing HOX-1 expression, exhibiting pro-oxidative effects and reducing cell migration. Molecular simulations show that heme binds favorably to HOX-1, followed by the other compounds, primarily through van der Waals and electrostatic forces. However, only van der Waals forces determine the H-Styr complexation. These interactions, influenced by metalloporphyrin characteristics, provide insights into HOX-1 regulation and ROS generation, potentially guiding the development of breast cancer therapies targeting oxidative stress.

## Introduction

Multiple cellular reactive oxygen species (ROS) serve as protumorigenic signals at certain levels. Different cancer cells exhibit redox homeostasis and can adapt to the cytotoxic ROS levels by increasing their antioxidant capacity.^1,2^ This optimization of ROS-driven proliferation and cell survival aims to inhibit ROS-triggered apoptosis and ferroptosis.^3,4^ For example, ROS promotes breast cancer cell proliferation, invasion, and angiogenesis by inducing specific metabolic pathways.^5^ Therefore, a proposed approach for breast cancer treatment involves using pro-oxidant agents to induce excessive ROS production and counteract cancer cells’ antioxidant capability.^6^ Some cancer therapies, like radiotherapy and certain chemotherapeutic agents, target the overproduction of reactive oxygen and nitrogen species, leading to oxidative stress-induced tumor cell death.^7,8^ Combining pro-oxidant agents with conventional cancer therapeutics has been suggested for more effective treatments, although further investigations is needed.^6^

Hemin, the coordinate complex of iron (Fe(III)) and protoporphyrin IX, is one of the porphyrin family used to treat acute intermittent porphyria.^9^ Due to its capacity to modulate diverse energy-related metabolic pathways, influence the tumor microenvironment, and enhance intracellular reactive oxygen species levels, heme (along with its oxidized form, hemin) has been recognized as a significant contributor to tumor progression.^10^ For instance, hemin was proposed as a chemopreventive agent, with a potency to interfere with various carcinogens in case of skin cancer.^11,12^ Similar roles were observed in the prostate,^13,14^ colorectal^15^ and lung cancers^16^ at specific hemin concentrations. However, high concentrations of dietary hemin stimulated intestinal tumorigenesis^17^ and colon cancer cell repopulation.^18,19^ In the case of breast cancer, hemin showed antitumor activities with modulation of cell migration and invasion rates through the modulation of certain pathways involved in the epithelial–mesenchymal transition.^20−22^

On a molecular level, hemin experiences specific electronic and chemical alterations, particularly in the presence of oxidizing agents like hydrogen peroxide (H_2_O_2_). These cause conformational alterations in the porphyrin ring, alongside a series of oxidoreductase cycles of the central Fe, with the generation of hydroxyl (HO^●^) and hydroperoxyl (HOO^●^) radicals via Fenton’s chemistry and Haber-Weiss reaction.^23,24^ These generated reactive species are responsible for the pro-oxidant activity of hemin, with implications in the progression of certain tumors at certain concentrations of hemin.^25−27^ Moreover, these excessively generated ROS induce ferroptosis of platelets,^28^ lipid peroxidation, and DNA damage toward the development of mutations.^29^ However, another strategy relies on the degradation of excess hemin via the action of heme oxygenases as the primary regulatory mechanism for intracellular hemin. Heme oxygenases catalyze the oxidative cleavage of the protoporphyrin IX ring of heme (and hemin), yielding biliverdin, carbon monoxide (CO), and labile iron.^30^ This reduces the cytotoxic effects of hemin and the accompanying oxidative stress, apart from the antioxidant actions of bilirubin, resulting from the reduction of biliverdin.^29^ Moreover, the released CO slows down the further release of heme from certain hemoproteins, besides its anti-inflammatory^31^ and anticancer activities.^32^ Nevertheless, other studies report a negative correlation between heme oxygenase-1 (HOX-1) activity and breast cancer progression.^33−35^ These variations can be related to the different types of breast cancer models studied and the mechanism of HOX-1 induction. These observations suggest the requirement for a controlled delivery method of low concentrations of hemin and/or the use of analogues with modulated pro-oxidant activities.

We have previously detailed the synthesis of various hemin derivatives achieved through conjugation to tyrosine using carbodiimide chemistry and to styrene via cross-metathesis reaction.^36,37^ Here, a cell-based investigation was conducted to assess the impact of hemin and its derivatives, hemin-tyrosine (H-Tyros), and hemin-styrene (H-Styr), on the migration of MDA-MB-231 triple-negative breast cancer cells. Subsequently, the influence of these compounds on HOX-1 protein expression and bilirubin production indicative of HOX-1 activity was evaluated. Given the alterations in hemin’s electronic properties upon conjugation, as previously reported,^36,37^ the effect of the tested compounds on intracellular ROS production and light generation in response to chemiluminescence reactions was also examined. Additionally, considering the death receptor and mitochondrial (intrinsic) pathways as the two main apoptosis signaling pathways,^38^ the expression of Caspase-3 and Poly [ADP-ribose] polymerase 1 (PARP-1), as well as mitochondrial functions, in response to the different compounds, were also studied. Furthermore, to comprehend the binding affinities between HOX-1 protein and heme as well as the different compounds, a molecular docking study followed by extensive molecular dynamics simulations was conducted. This comprehensive approach aimed to validate the interactions between metalloporphyrins and the HOX-1 protein and elucidate their subsequent intracellular effects.

## Results and Discussion

### Chemistry

**Figure 1:**
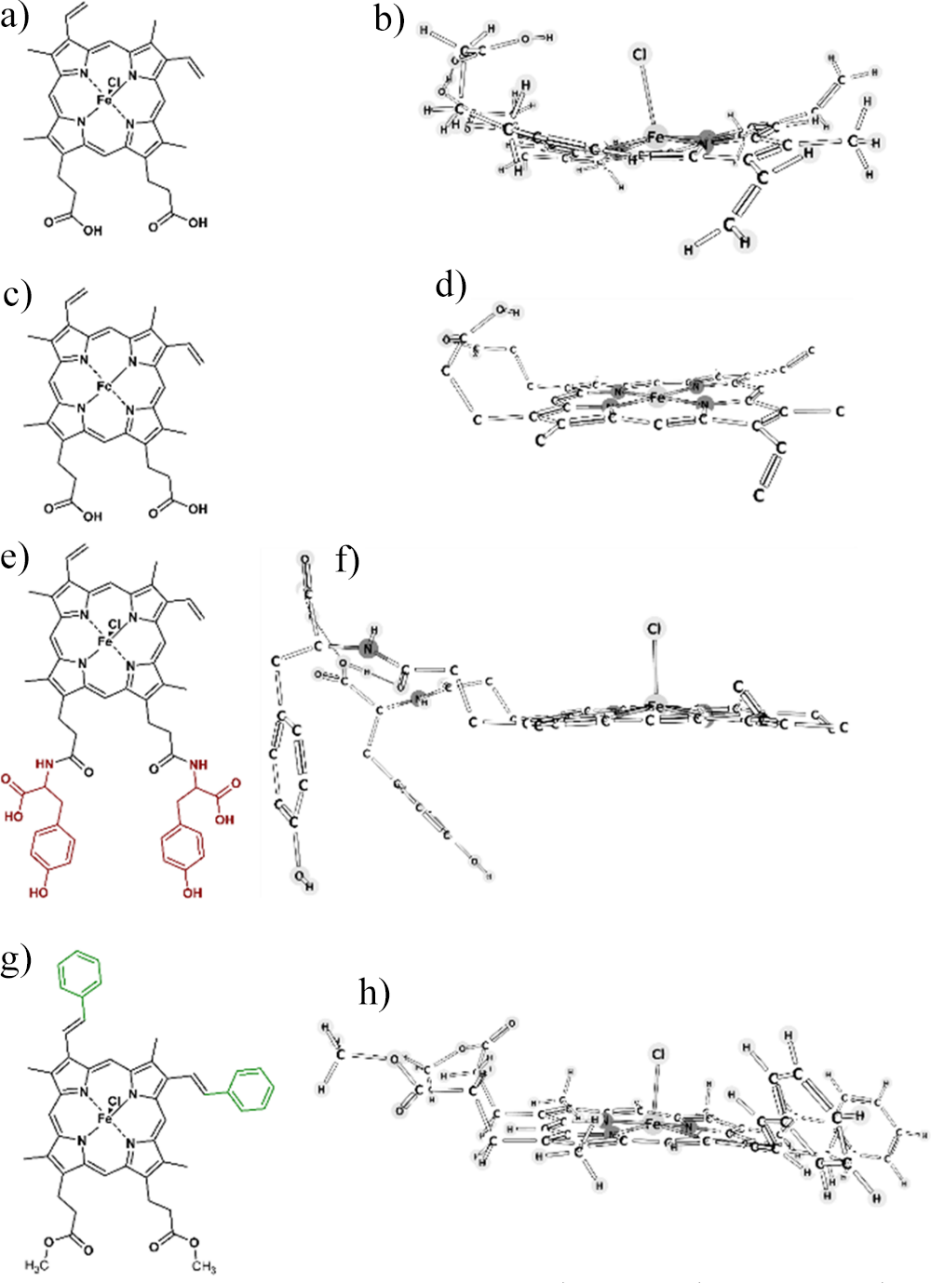
2D structure of hemin (a), heme (c), H-Tyros (e), and H-Styr (g), and the corresponding side view of the optimized 3D structures in (b), (d), (f), and (h), respectively.

The chemical structures of heme, hemin, H-Tyros, and H-Styr are shown in Figure 1, and the accompanying XYZ files are in Supporting Information (SI), Tables S1–S4. In humans, two isoforms of HOX exist, with several dynamic and structural differences.^39^ These are (1) the inducible form (HOX-1), which is generally expressed at low levels but induced by different regulators such as heat shock, lipopolysaccharides, oxidative stress and its substrate, heme (and hemin),^40^ and (2) the constitutive form (HOX-2), which degrades hemin under homeostatic conditions.^41^ Hence, this study focused on the roles played by hemin and its derivatives in modulating the expression of HOX-1 and employed its protein in the docking study. These calculations were carried out to predict the binding between HOX-1 and each tested compound to understand their effects on enzyme activity. The tested concentrations of compounds were proven to be cytocompatible based on the metabolic activity results, reported before.^36,37,42^

### Cell Migration

This was evaluated in MDA-MB-231 cells using the transwell migration assay and scratch assay. In the transwell assay, the tested compounds were diluted in fetal bovine serum (FBS)-containing medium placed in the lower chamber, and the number of cells that migrated toward this medium was quantified. As shown in SI, Figure S2a,b, only hemin at 4 μM significantly inhibited cell migration. However, at 8 μM, hemin had no effect, likely due to hemin aggregation, which we reported previously.^36^ H-Tyros enhanced migration at both tested concentrations, suggesting that it may induce certain chemical changes that promote cell movement (SI, Videos S4 and S5). Similarly, 4 μM H-Styr significantly enhanced migration, while the higher concentration had no effect. However, in the scratch assay, direct contact between the cells and the tested compounds inhibited their migration, preventing them from closing the gap (SI, Figure S2c). SI, Video S1 shows the change in the gap area in the case of untreated cells. The inhibitory effects were more pronounced at a higher concentration of 8 μM compared to the lower concentrations (SI, Figure S2c). Among the compounds, hemin exhibited the strongest inhibitory effect on cell migration (SI, Videos S2 and S3), followed by H-Styr (SI, Videos S4 and S5). H-Tyros showed the least inhibitory effect (Videos S6 and S7). It is noteworthy mentioning that the tested concentrations of these compounds were proved to be cytocompatible as we reported before.^37,42^

### Variability in Compound Effects on HOX-1 Expression Induction and Bilirubin Generation

**Figure 2:**
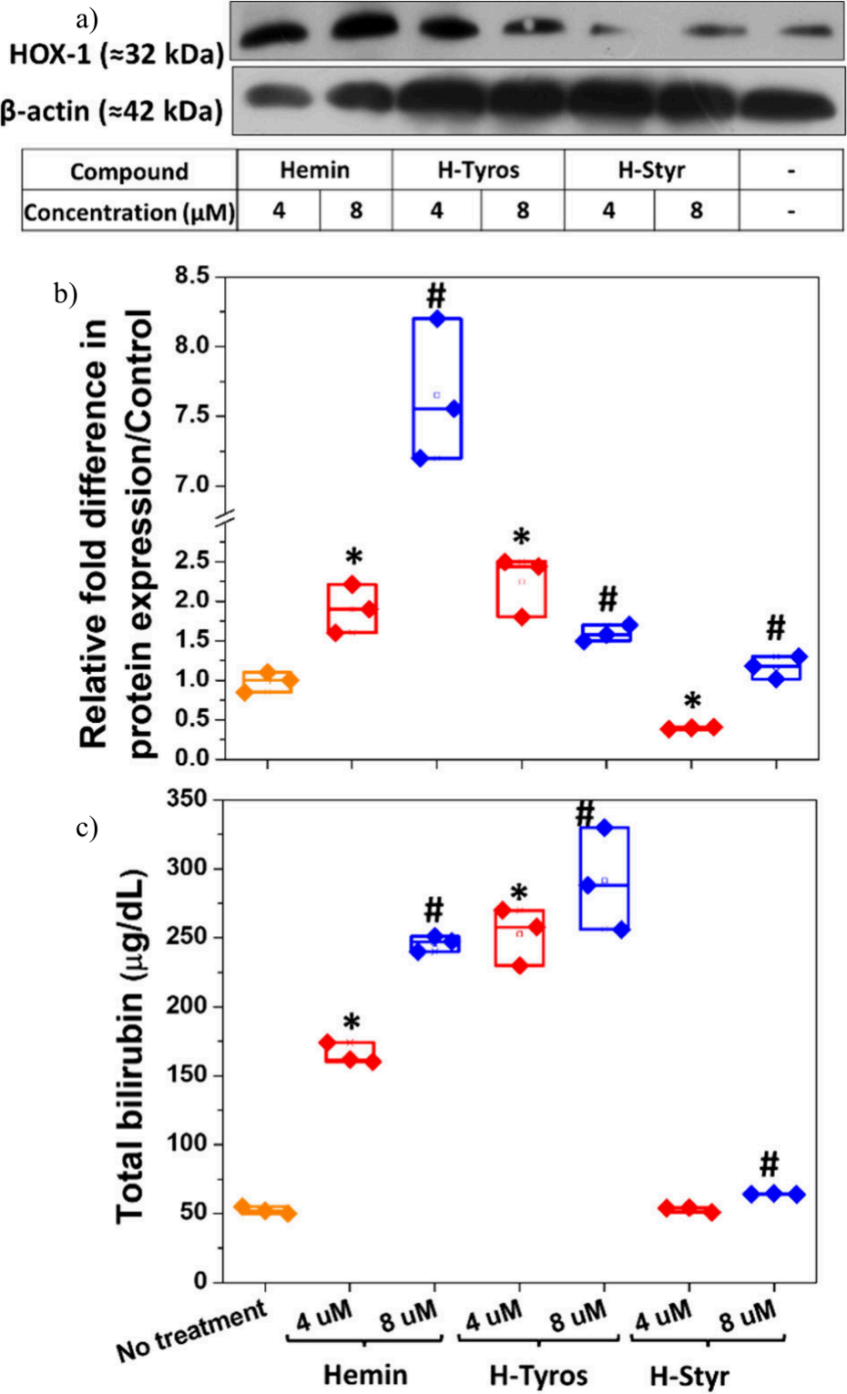
Compounds cause changes in the expression of HOX-1 with turnover to bilirubin in MDA-MB-231 cells. (a) Immunoblots after cell treatment with 4 and 8 μM hemin, H-Tyros, and H-Styr utilizing 5 μg of proteins/well. (b) Relative quantification of HOX-1. (c) Bilirubin extracted from cells upon different treatments. Results are presented as mean ± SD, *n* = 3, *, *P* < 0.05 versus the untreated cells in the case of 4 μM treatment; ^#^, *P* < 0.05 versus the untreated cells in the case of 8 μM treatment using a two-tailed unpaired Student’s *t* test. This experiment was repeated in duplicate.

Hemin is one of the main inducers of HOX-1 expression, causing heme/hemin degradation and forming bilirubin, carbon monoxide, and iron ions.^44−46^ We previously studied the hemin-inducing effects for HOX-1.^22^ However, here, a comparison of the effects of different concentrations of hemin and its derivatives on the HOX-1 expression was performed. At 4 μM, hemin and H-Tyros increased HOX-1 expression significantly (1.9 ± 0.3 and 2.4 ± 0.2, respectively), which decreased significantly in H-Styr-treated cells (0.4 ± 0.08) (Figures 2a,b).

However, while 8 μM Hemin and H-Styr caused a significantly high increase in levels of HOX-1 expression (7.5 ± 0.7 and 1.18 ± 0.12, respectively), these levels decreased at 8 μM H-Tyros (1.6 ± 0.18) compared to the corresponding lower tested concentrations. These results generally refer to multiple effects of the tested compounds on the expression of HOX-1, with a possible further influence on their catabolism once internalized into the cells. It should be emphasized here that the cell treatment was performed in FBS-free medium to allow for cellular uptake of the different compounds and prevent their scavenging by FBS constituents. We previously demonstrated that hemin has lower cellular uptake in FBS-containing medium compared to FBS-free medium.^42^

Similarly, the different compounds showed alterations in the levels of bilirubin, detected following the extraction of proteins from treated cells. Bilirubin generation was measured as a marker for the activity of HOX-1, resulting from intracellular heme degradation. As a constant amount of protein (10 μg) was used for bilirubin detection, the results are expressed as μg of bilirubin per dL of assay solution. Homogenization of cells was done using only deionized water to avoid interference from other homogenization reagents, resulting in a low protein yield per sample. To optimize the minimum protein concentration suitable for bilirubin detection, efforts were made to achieve an absorbance higher than 0.1, conducive to colorimetric detection. Consequently, 10 μg of protein was chosen for all measurements, yielding absorbances within the range of 0.1–0.35.

Like their effects on HOX-1 expression, 4 μM hemin and H-Tyros increased bilirubin generation significantly (165 ± 7.6 and 253 ± 20 μg/dL, respectively) but with no alterations in the case of H-Styr-treated cells (53 ± 1.8 μg/dL) compared to the untreated cells (Figure 2c). Moreover, bilirubin production increased at the higher concentrations reaching 246 ± 5.6, 291 ± 37 and 64 ± 3 μg/dL, in hemin, H-Tyros and H-Styr-treated cells, respectively. These results confirm the correlation between HOX-1 expression and heme/hemin degradation with generation of bilirubin. However, this correlation was postulated to be cell specific, and the induction of HOX-1 is not necessarily accompanied by heme degradation under oxidative stress.^45,46^ Moreover, considering the necessity of binding between the iron–metalloporphyrin and HOX-1 protein for its activation, this binding was explained later by MD and molecular docking simulations.

### Expression of Procaspase-3 and Degradation of PARP-1 in Response to the Different Compounds

**Figure 3:**
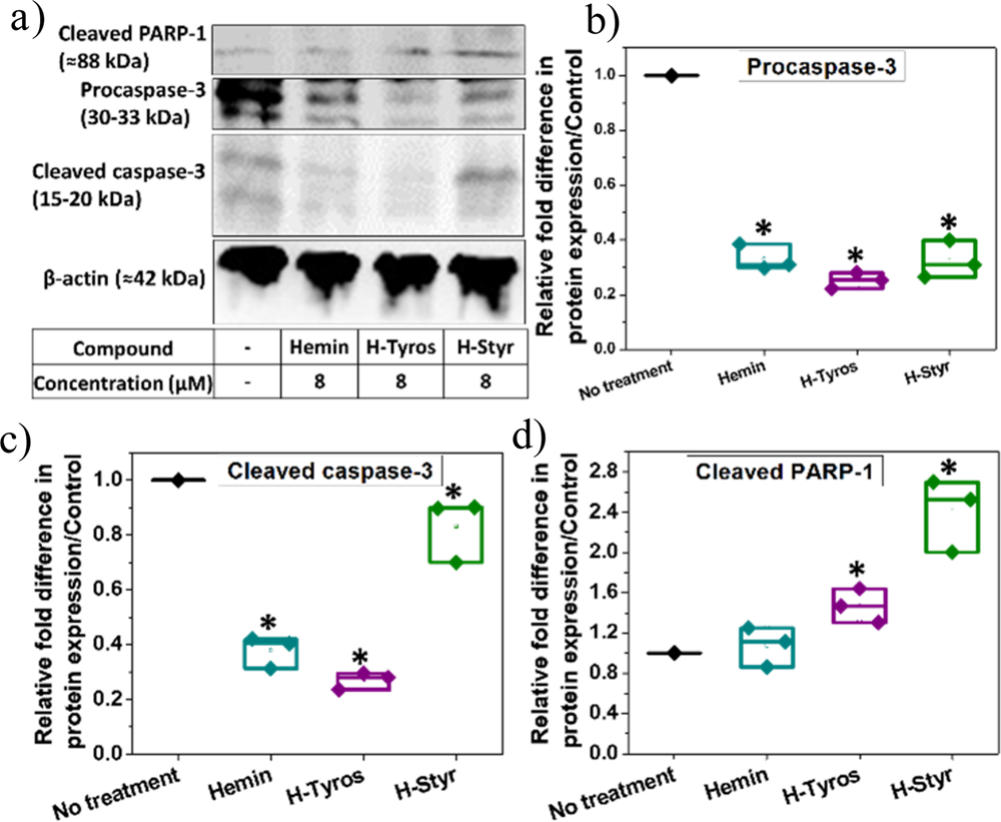
Compounds cause changes in the expression of apoptosis markers in MDA-MB-231 cells. (a) Immunoblots after cell treatment with 8 μM hemin, H-Tyros, and H-Styr utilizing 20 μg of proteins/well. (b–d) Relative quantification of procaspase-3, cleaved caspase-3, and cleaved PARP-1, respectively. Results are presented as mean ± SD, *n* = 3, *, *P* < 0.05 versus the untreated cells using a two-tailed unpaired student’s *t* test. This experiment was repeated in duplicate.

. Caspase-3 and PARP-1 are two major markers of cells undergoing apoptosis, with the latter serving as a substrate for active caspase-3.^47,48^ Notably, the downregulation of caspase-3^49^ and the overexpression of PARP-1^50^ have been reported as markers of breast carcinogenesis. The different compounds significantly decreased the expression of procaspase-3, without significant differences among their effects (Figure 3a,b). Similarly, the expression of cleaved caspase-3 decreased under the different treatments with H-Styr showing the least inhibitory effects (Figure 3c). A negative correlation was reported between the expression of HOX-1, mediated by hemin, and caspase-3 expression.^51,52^ These observations explain our findings and indicate that decreased expression of caspase-3 is a downstream effect of enhanced HOX-1 expression in the tested MDA-MB-231 cells. However, the exact mechanism and activity of caspase-3 were not studied in the current work. Similar changes in the expression of caspase-3 were also reported in MDA-MB-231 cells treated with genistein,^53^ quercetin,^54^ and extracts of *Cyperus rotundus* rhizomes,^55^ proven to have antitumor properties. Caspase-3 is responsible for the cleavage of PARP-1, generating 85 and 24 kDa fragments.^56,57^ The expression and accumulation of cleaved PARP-1 are considered to be hallmarks of apoptosis. Here, both H-Tyros and H-Styr significantly enhanced the expression of cleaved PARP-1, while hemin showed no effects (Figure 3d). Although hemin has been shown to increase PARP-1 cleavage in prostate cancer cells,^58^ this was not observed in MDA-MB-231 cells, which may indicate a lower sensitivity of these cells to hemin regarding PARP-1 cleavage, and a need for higher concentrations of hemin. However, the cells were more sensitive to the same concentrations of the other compounds.

### The Different Compounds Induce Various Alterations in the Mitochondrial Functions

**Figure 4:**
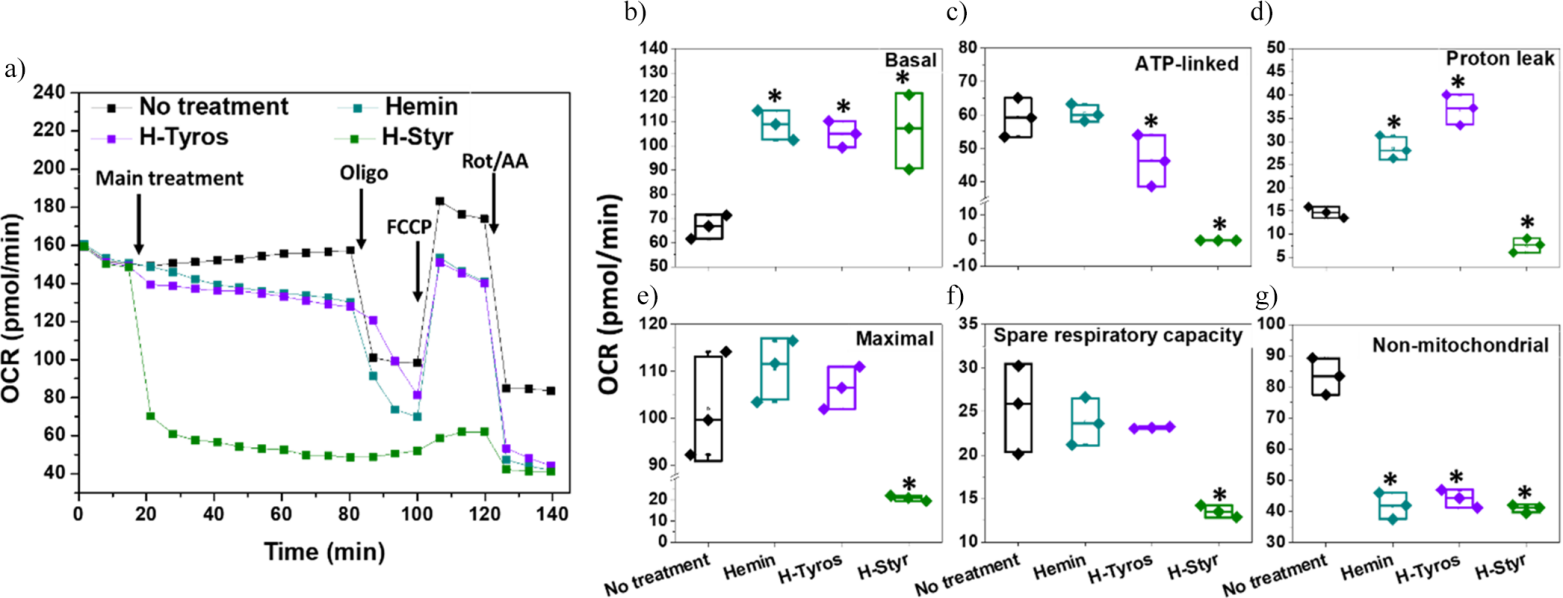
Impact of 1 h of treatment with tested compounds on the mitochondrial function of MDA-MB-231 cells, assessed using the Mito Stress Test. (a) A typical kinetic plot illustrates the alterations in OCR values subsequent to the acute injection of 8 μM hemin, H-Tyros, and H-Styr to cells, followed by 1 h of incubation, and then proceeding with the standard assay procedures. The specific parameters of mitochondrial function were analyzed: (b) basal, (c) ATP-linked, (d) proton leak, (e) maximal respiration, (f) spare respiratory capacity, and (g) nonmitochondrial OCR. Results are expressed as mean ± SD; *n* = 3, *p < 0.05 compared to the untreated cells using a two-tailed unpaired student’s *t* test. This experiment was replicated twice.

Mitochondrial respiration enables aerobic organisms to achieve higher energy production efficiency.^59^ However, during mitochondrial ATP synthesis, electron leakage to O_2_ occurs, leading to the production of reactive oxygen species (ROS). We previously discussed the influence of hemin on the mitochondrial functions of MDA-MB-231 cells.^59^ Here, these effects were compared to those of the hemin derivatives. The experiment started with three basal rate measurements taken prior to the injection of each compound. It should be emphasized that the group with no treatment refers to the negative control group, where only DMSO-containing medium was used, resulting in a final DMSO concentration of 0.008%. Generally, the OCR readings decreased once each compound was injected to the wells, with the highest drop level detected in case of H-Styr (Figure 4a). However, the other three compounds showed slight variations from the OCR in the case of untreated cells. These generally indicate the transient suppression of basal respiration. Nevertheless, following the treatment for 1 h, the calculated basal OCR increased compared to the untreated cells, with similar levels among hemin (96.3 ± 8 pmol/min) and H-Tyros-treated cells (98 ± 3.7 pmol/min) (Figure 4b). The hemin treatment of MDA-MB-231 cells did not cause significant changes in the ATP-linked OCR, which decreased due to the other compounds, particularly in H-Styr-treated cells (0.065 ± 0.003 pmol/min; Figure 4c). Reflected by its inducing effects for HOX-1 expression and activity, hemin at concentrations higher than 2 μM decreased the ATP production and maximal respiration while increasing the proton leakage in murine embryonic fibroblasts.^43^ However, here, owing to the short period of cell incubation with the different compounds, no significant changes in ATP-linked OCR due to hemin were detected. Nevertheless, the other compounds decreased the levels of these OCR levels.

While H-Styr decreased the proton leak (7.67 ± 1.5 pmol/min), the other compounds increased its levels, with the maximum enhancement detected in the case of H-Tyros (37.2 ± 1.8 pmol/min) (Figure 4d). These effects indicate some inhibitory effects for olig activity, with a possible decrease in the mitochondrial membrane potential. Olig activity mediates its effects by inhibiting ATP synthase, which decreases electron flow through the electron transport chain. This leads to a reduction in mitochondrial respiration and consequently lowers cellular ATP production. Hemin (111.7 ± 7.8 pmol/min) and H-Tyros (106.5 ± 4.5 pmol/min) did not alter the maximal respiration significantly, which dropped in the case of H-Styr-treated cells only (20.8 ± 1.1 pmol/min) (Figure 4e), indicating a severe inhibition of mitochondrial respiration due to H-Styr. These changes were accompanied by a decreased spare respiratory capacity due to H-Styr (13.5 ± 0.7 pmol/min), but with no changes in hemin and H-Tyros-treated cells (Figure 4f). Nevertheless, all compounds significantly inhibited the nonmitochondrial respiration (Figure 4g). These results generally refer to various effects of these tested compounds on the different mitochondrial functions, with hemin and H-Tyros showing similar interactions with the mechanisms involved. Moreover, while the generation of ROS was reported as a main factor responsible for the mitochondrial dysfunction,^60,61^ this was not the case at the tested concentrations of the different compounds, considering the different ROS levels generated as explained in the following section. These effects can be due to particular effects of the tested compounds themselves, which is outside the scope of this study.

### Diverse Compound-Induced ROS Generation

**Figure 5:**
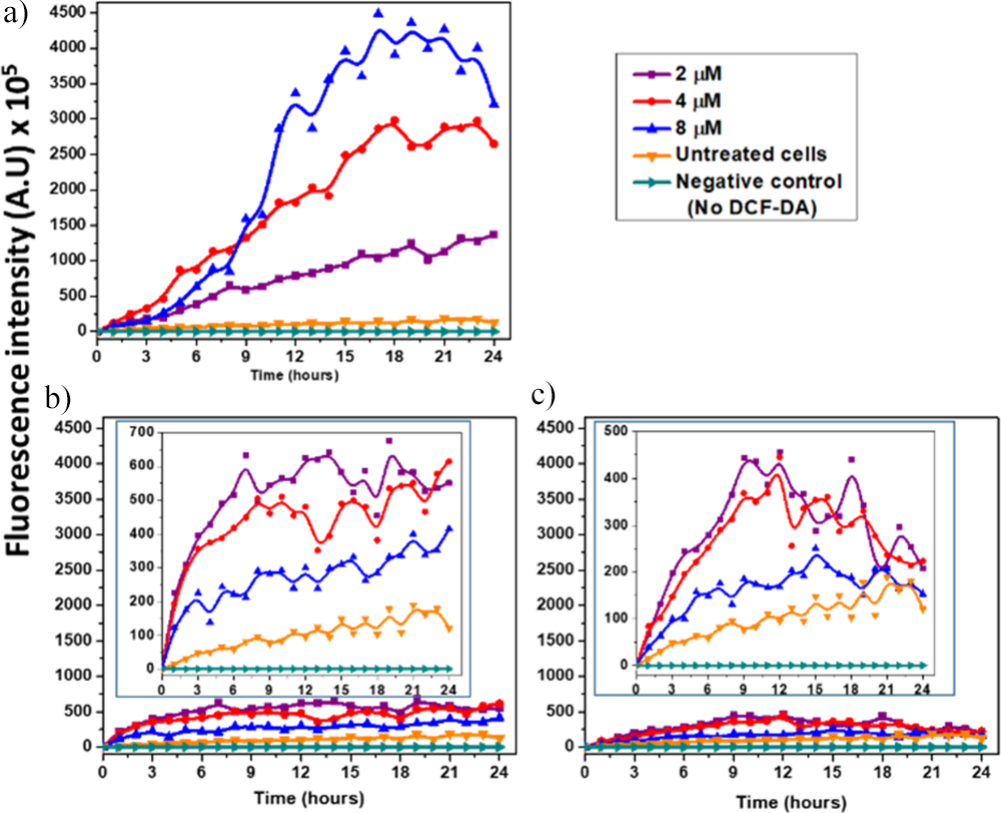
Kinetics of the changes in intracellular ROS levels detected by DCF-fluorescence in MDA-MB-231 cells treated with either hemin (a), H-Tyros (b), or H-Styr (c) at the concentrations 2 (solid purple line), 4 (solid red line), or 8 μM (solid blue line). The fluorescence due to DCF in untreated cells and without any DCF treatment is expressed by orange and cyan colors, respectively. The cells were treated with DCF-DA for 45 min and then photographed after the different treatments were added using the real-time Incucyte imaging system. Results are presented as mean fluorescence intensity, *n* = 3.

The ROS detection study was initiated by testing the metabolic activity of MDA-MB-231 against different concentrations of *tert*-butyl hydroperoxide (tBuOOH) and H_2_O_2_ as radical initiators. This was followed by finding out the optimum concentration of 2′,7’–dichlorofluorescein diacetate (DCF-DA) for detection of intracellular ROS. These findings are summarized in Supporting Information. The optimal detection of intracellular ROS was performed in the absence of FBS to avoid its fluorescence-quenching effects. Treatment of cells with different hemin compounds in a FBS-free medium induced the generation of different levels of ROS. This depended on the type of compound and its concentration. A distinct difference in fluorescence intensity was detected between the DCF-DA treated and untreated cells (Figure 5; SI, Videos S8 and S9). However, this intensity significantly increased with the different treatments, with fluorescence accumulation over the 24 h of cell imaging. The highest levels of ROS were generated after hemin treatment (Figure 5a; SI, Videos S10, S11, and S12), followed by H-Tyros (Figure 5b; SI, Videos S12, S13 and S14), with H-Styr causing the lowest levels of ROS production (Figure 5c; SI, Videos S15, S16, and S17). Moreover, in hemin, the ROS levels were directly proportional to the tested concentration, while, in H-Tyros and H-Styr, this was an inverse relation. Of a note, although the different compounds showed similar inhibitory effects for procaspase-3 expression (Figure 3b), this was not correlated with similar inducing levels for intracellular ROS generation.

These differences relate to two mechanisms governing ROS generation induction in response to these compounds. The first relates to the oxidation of these metalloporphyrin compounds inside the cells with reduced H_2_O_2_ and generation of HO^●^ radicals via a Fenton-type reaction.^62,63^ We reported before the susceptibility of these compounds toward the oxidizing effects of H_2_O_2_.^36^ Here, hemin showed a higher oxidation potential, followed by H-Tyros, and finally, H-Styr. This changing trend in the oxidation potential was also observed when these compounds were in complexation with BSA as a model protein. This makes hemin a more potent catalyst of oxidative injury due to H_2_O_2_. Notably, these ROS-inducing effects were independent of the cellular uptake of these compounds, where despite the higher uptake of H-Styr than both other compounds,^36^ showed the least enhancement in the generation of intracellular ROS.

The second factor is each complex’s magnetic properties and electronic configuration. As we reported before, the iron atom in hemin has a characteristic high spin, and H-Styr has a low-spin Fe(II)–organic radical configuration.^36^ Moreover, H-Tyros molecules bind more water molecules, with magnetic properties similar to those of H-Styr at certain conditions. Hence, the susceptibility of H-Tyros and H-Styr to oxidation and their tendency to generate intracellular ROS were lower than that of hemin. In addition, this low-spin Fe(II) character seems to compromise the main steps of the oxidation of H-Tyros and H-Styr, which explains the lower levels of ROS generation detected with an increase in their concentration. In support of our results, another study reported that hemin-induced ROS production relates to the generation of ferryl and perferryl radicals from the hemin (Fe(III)/H_2_O_2_ interactions.^64^ These reactions will become more favorable in the presence of ferryl heme (Fe(III)-PPIX), particularly at the low cellular concentrations of H_2_O_2_ responsible for the oxidation of heme (Fe(II)-PPIX) for further formation of ferryl heme. To substantiate this hypothesis, the luminescence produced in response to H_2_O_2_/luminol mixtures was measured in the presence of one of the tested compounds, indicating chemiluminescence (CL) reactions. In brief, hemin showed the highest catalytic efficiency for these reactions and enhanced luminescence kinetics, followed by H-Tyros and finally H-Styr, exhibiting the lowest luminescence yield in different solutions (SI, Figure S4). Details of this study are explained in the Supporting Information.

**Scheme 1:**
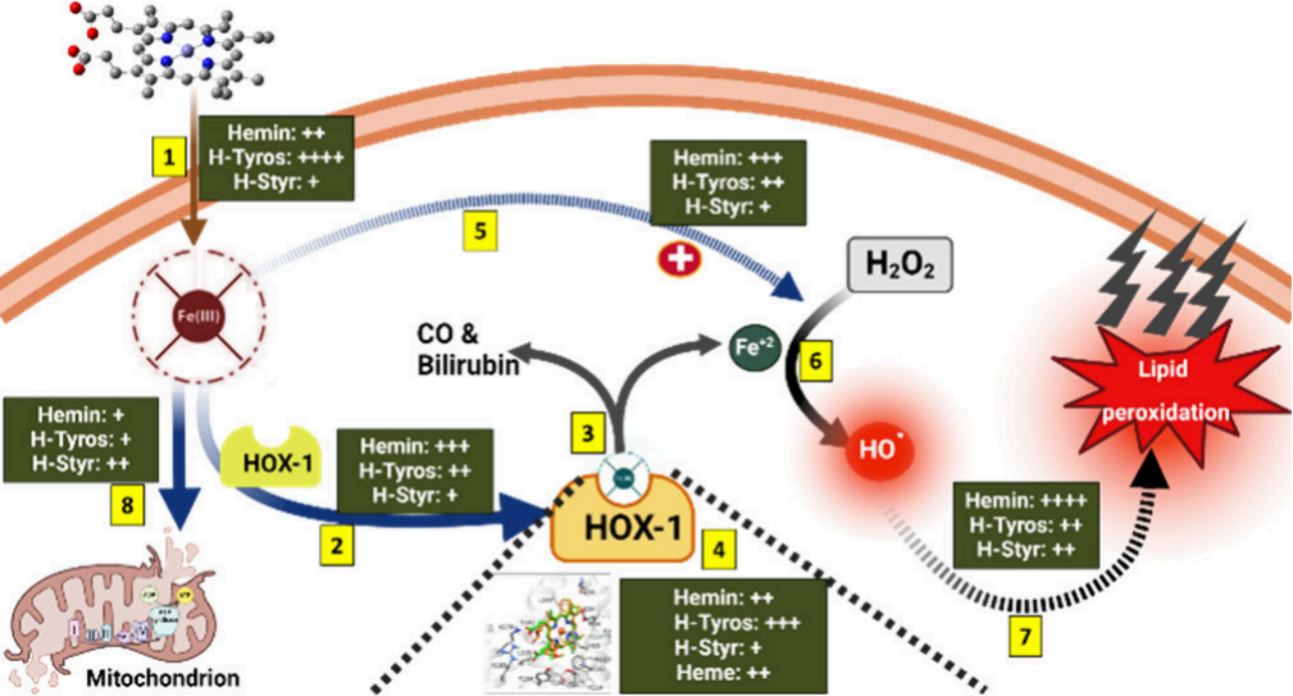
Cellular Internalization of the Tested Compounds and Their Influence on Heme Oxygenase Activity, ROS Generation, and Mitochondrial Functions (1) Each compound has a specific affinity to permeate through the cell membrane, with varying enhancing efficiencies for HOX-1 expression (2), which upon activation, catalyzes the generation of CO, bilirubin and Fe^+2^ ions (3). (4) For activation of enzyme functions, each compound should bind to the HOX-1 protein with different affinity compared to heme. (5) Upon cellular uptake, all compounds have different affinities as catalysts for the Fenton reaction and the production of intracellular ROS, particularly the HO^●^ species, which is also catalyzed by the Fe^+2^ ions (6). The excessive production of these reactive species can induce lipid peroxidation and possible ferroptosis (7). (8) Additionally, the tested compounds have different effects on the mitochondrial respiration and its dysfunction. Insets in green boxes: Comparison of the influence of the tested compounds on the described intracellular reactions. Schematic created with BioRender.com.

The third factor is dependent on the activity of HOX-1. As explained before, hemin has the highest inducing effects for HOX-1 expression, compared to both H-Tyros and H-Styr, with 4 μM H-Tyros having higher efficiency than 8 μM. This enzyme neutralizes the physiological effects of the free heme/hemin molecules and protects against their inducing effects for intracellular ROS generation.^65,66^ However, the imbalance between the enzymatic degradation of hemin and its pro-oxidation effects will lead to ROS generation. Coló et al., 2023 reported an overexpression of ferritin in hemin-treated breast cancer cells.^21^ This iron-related protein protects cells against the pro-oxidant effects of iron through its storage. Hence, with the increase in concentration of these compounds, disturbance of the balance between iron release and storage would happen with a possible increase in the intracellular ROS levels. That will, in turn, induce apoptosis,^67^ in addition to ferroptosis due to the accumulation of oxidatively damaged phospholipids.^68^ To summarize, while hemin showed the highest inducing effects for HOX-1 expression, its highest oxidizing potential is responsible for the most intensive ROS generation. In spite of the antioxidative activity of the generated bilirubin, this could not protect against the oxidative stress in response to hemin. However, considering the similar H-Tyros-induced HOX-1 activity at 4 μM, with a relatively higher rate of bilirubin production and its lower oxidation potential, this compound can be an ideal alternative for overcoming the pro-oxidative effects of hemin. Nevertheless, H-Styr showed the lowest effects toward enhancing HOX-1 expression, bilirubin production, and ROS generation. A summary of these events is shown in Scheme 1. Of a note, we previously discussed the differences in cellular uptake of the tested compounds.^36,37^

### Molecular Docking Studies

**Figure 6:**
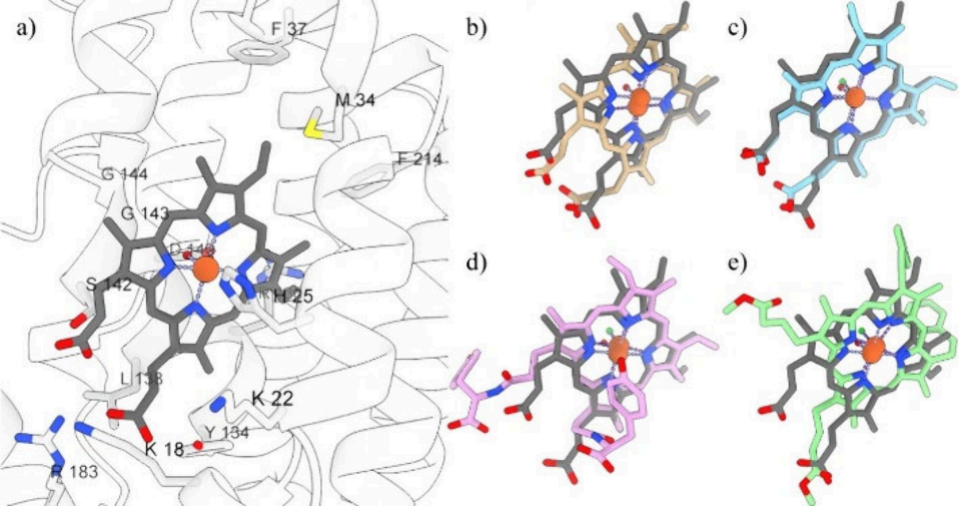
(a) Illustration of the HOX-1 crystallographic structure (PDB 1N45), depicting its heme substrate (gray color) and important residues. (b,e) Superposition of the most favorable conformations calculated from molecular docking relative to the crystallographic heme (gray) of (b) heme (brown), (c) hemin chloride (cyan), (d) H-Tyros (magenta), and (e) H-Styr (lime) derivatives. Fe(III), water, and Cl are displayed as orange, red, and green spheres. Color-coding is maintained throughout the manuscript.

HOX-1 is a 32–33 kDa membrane-bound protein composed of 288 amino acid residues and binds to heme, which works as its prosthetic group and the substrate.^69^ The mechanisms for three oxygenation reactions, catalyzed by HOX-1 for heme (Fe(II)-PPIX) and hemin (Fe(III)-PPIX) were previously explained.^70^ The compounds considered were docked to HOX-1, with their lowest energy binding modes relative to those of the crystallographic heme presented in Figure 6, along with the associated binding affinities (Table S5). Most HOXs target the hydroxylation of the α-meso carbon in heme.^71^ Heme is sandwiched between the proximal and distal helices of the protein structure with the α-meso edge of heme facing many hydrophobic residues (Figure 6a). These are phenylalanine 207 (F^207^) and 214 (F^214^), methionine 34 (M^34^), and histidine 25 (H^25^), with the latter acting as axial heme ligand.^72^ No relative change in these residues among heme (Figure 6b), hemin (Figure 6c), H-Tyros (Figure 6d) and H-Styr (Figure 6e) within the HOX-1 matrix was observed.

Specifically, from the binding modes obtained, it is observed that heme’s position is successfully reproduced, with a RMSD between the crystallographic and calculated coordinates equal to 1.3 Å. The porphyrin ring of all hemin derivatives was calculated to occupy the same position with heme adopting similar orientations, with the position the Cl atom bound to the central Fe(III) atom toward the aspartic acid residue 140 (D^140^) overlapping in position with the crystallographic water molecule. D^140^ was reported as a central residue for controlling the hydrogen-bonding network within the HO distal pocket toward optimal HOX-1 functionality and the first step of heme oxidation into α-*meso*-hydroxy heme.^73,74^ Moreover, the Cl atom is shifted toward the glycine residue 143 (G^143^) in all hemin derivatives. G^143^ is HOX-1’s closest residue to heme, as well as with G^139^ which is within H-bonding with the distal water ligand and interacts with oxygen in the oxyheme complex.^75^ In contrast to the β, γ, and δ meso carbons, the α-meso carbon is pointing toward the back of the active site pocket with less steric crowding, making it the only available carbon for the hydroxylation reaction.^76^ However, this mechanism is different from the nonenzymatic oxidation of heme, where the four meso carbons have nearly equal susceptibility to the oxidation.^77^ On the opposite side of the heme pocket, M^34^, F^37^, and F^214^ form a hydrophobic wall opposite the heme α-meso edge.^75^ Docking calculations showed that all hemin derivatives adopt conformations with their α-carbons pointing to these residues, while H-Styr was found to have its bulky styrene substituents buried close to these residues. The β-edge of all ligands was in proximity to tyrosine 134 (Y^134^) and threonine 135 (T^135^), with the direction of the heme/hemin propionates tilting toward the aromatic residue of Y^134^. This solvent exposed HOX-1 cavity region offers the possibility of a number of ionic/H-bonding interactions between the heme propionates and nearby side chains are important for orientating the heme in the active site;^75^ H-Tyros tyrosine and H-Styr methyl hexanoate substituents are also extended toward these residues.

HOX-1 catalyzes heme oxidation via O_2_ and NADPH-cytochrome P450 reductase-dependent mechanism.^30,78,79^ The lysine residues 18 (K^18^), 22 (K^22^), 179 (K^179^); arginine residues 183 (R^183^) and 198 (R^198^); and glutamic acid residues 19 (E^19^), 127 (E^127^) and 190 (E^190^) in HOX-1 structure contribute to its binding of cytochrome P450 reductase;^69^ docking calculations predict proximity of the hemin derivatives to these residues. R^183^, K^18^ and K^22^ were proven to have stabilizing functions for heme in the active site.^75^ However, only R^183^ was identified by docking calculations, which surrounds the propionate residues in heme and hemin and binds to each derivative via possible ionic and H-bonding, holding them within the HOX-1-matrix. Moreover, due to the occupation of the terminal propionates in H-Tyros via conjugation to tyrosine residues, these stabilizing effects of R^183^ were nearly absent, with an increased distance between this residue and these terminal groups. The serine residue 142 (S^142^) of the distal helix stabilizes the distortion of the distal helix through H-bonding with several peptide residues such as G^143^, leucine residues 138 (L^138^), and 141 (L^141^), and E^145^ and K^179^. The S^142^–L^138^ interact via hydrogen bonding between the −OH side chain of the former and carbonyl oxygen atoms of the later residue. Both heme/HOX-1 and hemin/HOX-1 were similarly placed relative to these residues, while H-Tyros and H-Styr were slightly reoriented away from the terminal chain in L^138^.

Overall, according to molecular docking calculations, heme and hemin possible H-bond formation is observed with K^18^, K^179^, R^183^ and close van der Waals contacts are observed with, E^29^, T^135^, Y^134^, R^136^, L^138^, V^146^, L^147^, and F^207^. One of the H-Tyros tyrosine moieties is forming a possible hydrogen bond with R^183^, while the other is facing the imidazole ring of H^25^, with close hydrophobic contacts observed with F^214^, K^22^, E^29^, G^143^, Q^145^, V^146^, A^175^, T^176^, and N^210^. Lastly, H-Styr methyl hexanoate is H-bond-distance with the backbone of V^146^, while the second methyl hexanoate is in H-bond proximity to R^183^ and Y^134^. The styrene moieties are directed inward facing the M^34^, F^37^ and F^214^ residues, as well as R^136^ and D^140^; R^136^ is facing one of the styrene rings. Other close contacts include A^28^, F^37^, Q^38^, L^138^, S^142^, G^143^, L^147^, F^207^, N^210^ and T^135^. Comparison of hemin derivatives’ binding affinities relative to the reference heme substrate indicates similar favorable interactions with HOX-1 (SI, Table S5). Notably, H-Tyros displayed the highest binding affinity among the derivatives, surpassing both heme and hemin, while H-Styr ranked the lowest. Nevertheless, these results underscore the affinity of these derivatives toward HOX-1. Subsequent molecular dynamics simulations were carried out to further evaluate the calculated binding modes and investigate the mechanistic aspects underlying the interaction with HOX-1. This work aims to offer a comprehensive understanding and validation of the binding interactions of heme and hemin derivatives.

### Molecular Dynamics Simulations

**Figure 7:**
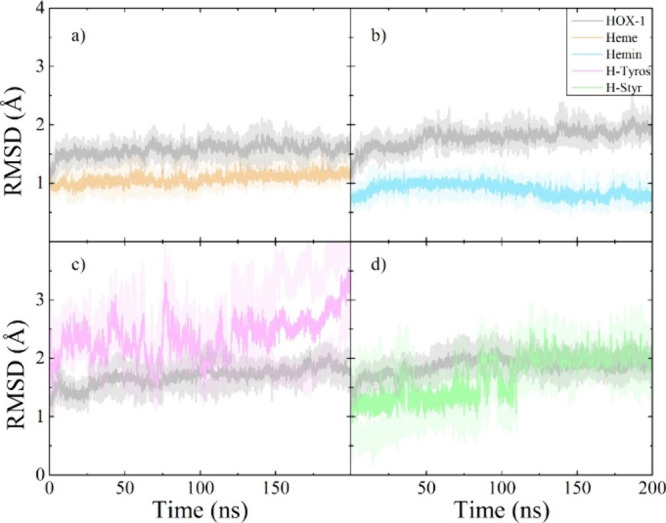
RMSD trajectory analyses of HOX-1 protein complexes with the (a) heme, (b) hemin, (c) H-Tyros derivatives, and (d) H-Styr derivatives. The solid lines depict the average RMSD value, while the shaded regions indicate the corresponding standard deviation from three independent simulations.

Subsequent to the molecular docking calculations, molecular dynamics (MD) simulations were conducted for hemin, H-Styr, and H-Tyros complexes with HOX-1. Binding free energies with the Molecular Mechanics Poisson–Boltzmann Surface Area (MM–PBSA) method were also computed, along with per-residue decomposition analysis, which provided useful insight on the individual contributions toward binding. Initially, analysis of the MD simulations revealed the structural stability of the complexes, as evidenced by the calculated Cα-based RMSD values of HOX-1 in its complexes with the derivatives considered. Specifically, RMSD analysis with respect to the crystallographic structure showed that binding of these compounds to the HOX-1 protein does not induce any notable structural effects, as it does not deviate significantly from the reference structure. This is also apparent by examination of each compound’s heavy atom RMSD fluctuation, which–apart from H-Tyros– appear very stable (Figures 7, and SI, Figure S5).

**Figure 8:**
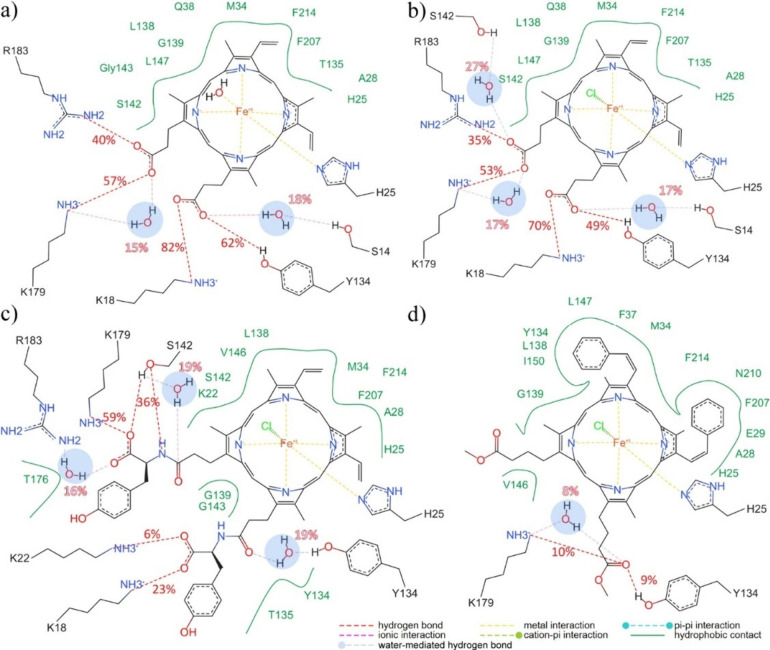
2D interaction diagrams of the (a) heme, (b) hemin, (c) H-Tyros, and (d) H-Styr derivatives in complex with HOX-1. Diagrams were prepared in PoseEdit from the centroid structures deduced by clustering conformational analysis of the MD simulation trajectories. Calculated percentage of direct hydrogen bond and water mediated hydrogen bonds occurrence are also illustrated.

Indeed, all derivatives maintained their relative positioning and arrangement as observed in the molecular docking results. The cluster centroid structures, representing each HOX-1 complex with these derivatives, illustrate the preservation of most interactions identified during molecular docking (SI, Figure S6). For comparison, SI, Figure S7, shows the centroid structure of the HOX-1 protein complex with heme, showcasing the water-mediated hydrogen bonding interaction involving the Fe(III) coordinated water and G^139^ and G^143^ residues. Hydrogen bond (HB) analysis supports observations regarding the hydrophobic nature of the interactions governing derivative complexation to HOX-1.^79^ While H-Styr shows no involvement in HBs with HOX-1, hemin chloride and H-Tyros facilitate HB formation with specific residues of the HOX-1 cavity entrance. Specifically, heme’s propionate groups engage in hydrogen bonding not only with K^18^, K^179^, and R^183^ but also with Y^134^ (Figure 8a, and SI, Figure S6a). The hydrogen bond with K^179^ is facilitated by water solvent molecules during 15% of the simulation time, similar to that with S^14^ (18% of the simulation time).

Furthermore, the Fe(III)-coordinated water acts as a bridge between the G^139^ and G^143^ residues (SI, Figure S7). Heme binding also involves van der Waals interactions with M^34^, L^138^, G^139^, V^146^, L^147^, and F^207^, highlighted as significant contributors to binding according to the per-residue decomposition analysis (SI, Table S6). Hemin adopts a similar conformation with a binding mode characterized by a comparable network of hydrogen bond residues, albeit with slightly reduced frequency (Figure 8b, and SI, Figure S6b). Moreover, the hydrogen bond with S^142^ is frequently mediated by a water solvent molecule.

One of the tyrosine moieties in H-Tyros establishes transient hydrogen bonds with K^18^ and K^22^, along with a water-mediated hydrogen bond with Y^134^. The other tyrosine moiety interacts less frequently with R^183^ via a water bridge, while its most stable hydrogen bond is with K^179^, alongside S^142^, where another water bridge was present for 19% of the simulation time (Figure 8c, and SI, Figure S6c). H-Tyros demonstrates notable hydrophobic contacts with residues K^22^, G^143^, Q^145^, V^146^, T^176^, F^204^, and F^214^, among the residues most conducive to complexation (SI, Table S6). Concerning H-Styr, it engages in limited and temporary hydrogen bonding interactions as its methyl hexanoate forms hydrogen bonds with R^183^ and Y^134^ for a brief period during the simulations (Figure 8d, and SI, Figure S6d). The styrene moieties are inwardly oriented, interacting with residues M^34^, F^37^, and F^214^, along with R^136^ and D^140^, with R^136^ situated close to one of the styrene rings. Additionally, notable residues favoring interaction include A^28^, M^34^, L^138^, S^142^, G^143^, L^147^, F^207^, N^210^, and T^135^ (SI, Table S6). In summary, there is a consistent trend of interaction with hydrophobic residues observed among heme, hemin, and H-Tyros, along with common hydrogen bonding interactions. Despite lacking significant hydrogen bonding, H-Styr interacts favorably with several favorable amino acids inside the HOX-1 active site.

Moreover, it is worth mentioning that based on the hydrogen bond analysis performed, hydrogen bonding between H^25^ and E^29^ is observed for most of the simulation time in the HOX-1 complexes with heme, hemin, and H-Tyros. This interaction involves primarily backbone (66%, 69%, and 74% occupancy, respectively) and to a smaller extent side chain atoms (9%, 17%, and 13% occupancy, respectively) and has been previously reported in the literature.^9^ Nonetheless, in the HOX-1 complex with H-Styr, this interaction was largely absent (Figure 8d), with occurrences of only 16% and 2% between backbone and side chain atoms, respectively, similar to an open active-site conformation.^9^

**Figure 9:**
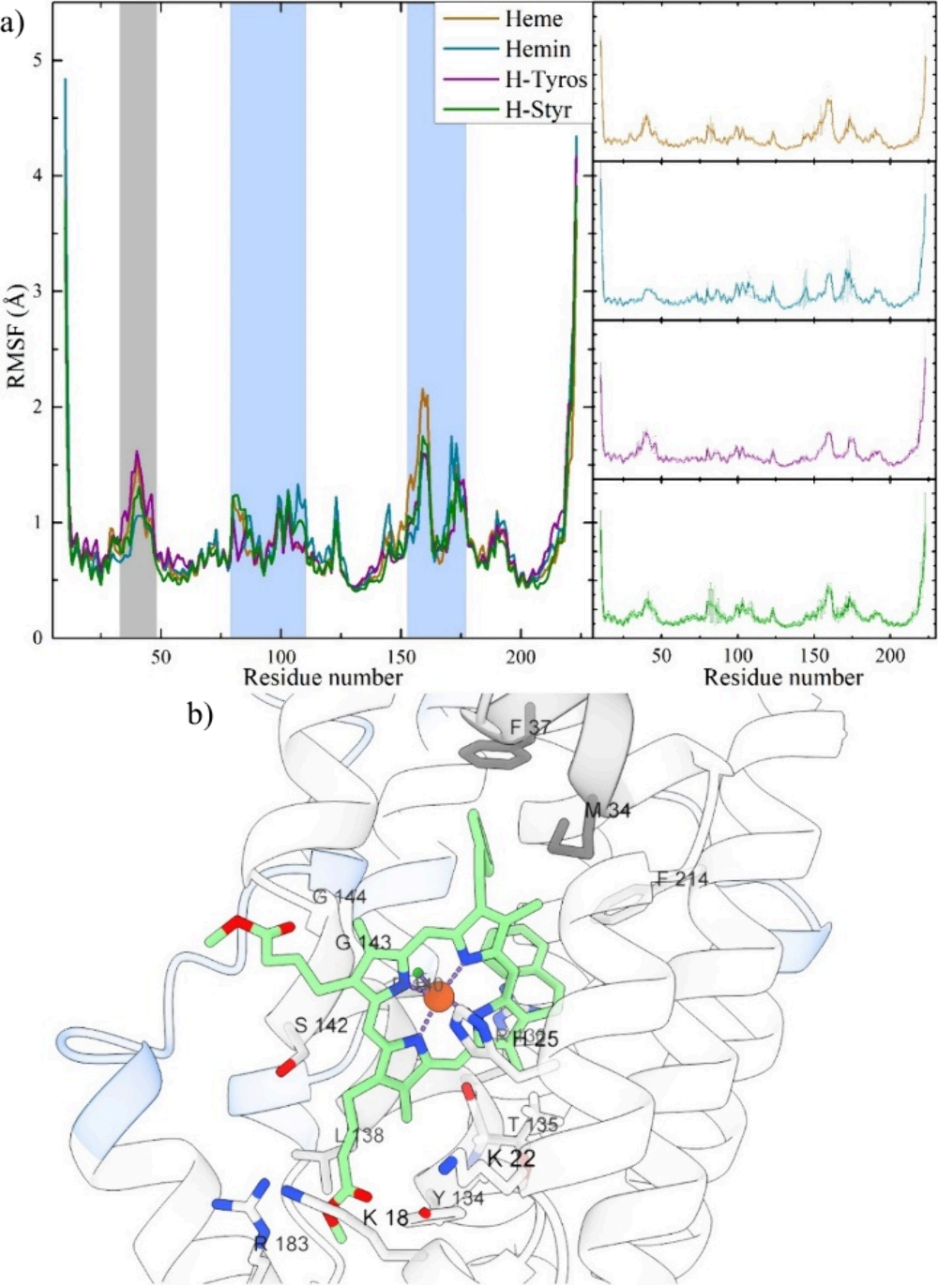
(a) RMSF analysis of the HOX-1 protein in complex with heme, hemin, H-Tyros, and H-Styr derivatives. The solid lines represent the average RMSF obtained from three independent simulations, as depicted in the insets, with error bars indicating statistical uncertainty. Protein regions with increased mobility are also highlighted (light gray and light blue). (b) Centroid structure of H-Styr in complex with HOX-1.

Regarding the HOX-1 protein structure, RMSF values indicate minimal mobility, suggesting limited structural changes in HOX-1, primarily observed in residues 34–46, 80–110, and 153–176 (Figure 9). The first region encompasses loop residues following the proximal helix, which, particularly G^40^, exhibit slightly more pronounced fluctuations in the heme and H-Tyros complexes, while appearing more stabilized in the hemin and H-Styr complexes (Figure 9, light gray). The latter two regions (Figure 9, light blue) encompass solvent-exposed residues found within the loops connecting helices situated between the proximal and distal helices.

The overall compactness of HOX-1 as determined by the radius of gyration (Rg) remains unchanged (SI, Figure S8), as well as the protein’s solvent-accessible surface area (*SASA*) in all complexes (SI, Figure S9). The substrate binding cavity is not significantly changed in the heme, hemin, and H-Styr complexes; however, it slightly expands within the margin of error to facilitate the bulkier H-Tyros substrate (SI, Figure S10). To better illustrate the placement of the derivatives considered within the cavity, the center of mass (COM) distances of residues D^140^, G^143^ and L^147^ and either the Fe(III) coordinated water molecule or Cl were computed. These residues are important in characterizing the apo and bound states of HOX-1 due to their relative positions.^9^ The calculated COM distances of H_2_O and Cl with L^147^ are similar in all complexes (SI, Figure S11 and Table S7). Notable discrepancies are observed in the case of H-Styr, where the Cl distance with residues D^140^ and G^143^ increases, which is indicative of a slight upward tilt of this molecule relative to that of the residues in question (Figure 8d).

Finally, MM–PBSA free energy calculations were conducted, indicating comparable binding energy values across all derivatives (Table 1). Remarkably, despite lacking hydrogen bond interactions, H-Styr exhibited a high binding free energy to HOX-1, emphasizing the hydrophobic nature of its binding. Conversely, binding with H-Tyros incurred a higher entropy penalty. The MM–PBSA analysis ranked the compounds in decreasing order of binding free energies to HOX-1 as follows: heme > hemin > H-Styr > H-Tyros. Van der Waals interactions play a substantial role in the binding enthalpy of all complexes, with nonpolar solvation effects following closely behind, while total electrostatics tend to disfavor complex formation. Furthermore, upon decomposing the binding energetics into individual terms, as shown in Table 1, it becomes apparent that the van der Waals (Δ*E*_vdW_) and the electrostatic component of the molecular mechanical energy (Δ*E*_elec_) contribute most significantly to the formation of all complexes except H-Styr.

**Table 1:** Energetic Analysis of the HOX-1 Complexes with Heme, Hemin, And Its Derivatives, H-Tyros and H-Styr, as Obtained with the Mechanics Poisson–Boltzmann Surface Area (MM–PBSA) Method (Uncertainties Are Included in Parentheses)

	compd			
energy (kcal mol^–1^)	heme	hemin	H-Tyros	H-Styr
Δ*E*_vdW_	–53.14 (0.04)	–56.67 (0.05)	–70.74 (0.06)	–80.29 (0.05)
Δ*E*_elec_	–241.94 (0.41)	–269.20 (0.47)	–205.93 (0.38)	–18.94 (0.09)
Δ*E*_MM,gas_	–295.07 (0.41)	–325.87 (0.48)	–276.67 (0.38)	–99.23 (0.10)
Δ*G*_PB_	251.13 (0.35)	286.38 (0.41)	239.46 (0.34)	61.69 (0.09)
Δ*G*_elec(tot)_^a^	9.19 (0.54)	17.18 (0.63)	33.52 (0.50)	42.75 (0.12)
Δ*G*_NP_	–8.42 (0.01)	–8.84 (0.01)	–11.02 (0.01)	–11.11 (0.01)
Δ*G*_solv_	242.71 (0.35)	277.54 (0.41)	228.43 (0.33)	50.58 (0.09)
Δ*H*	–52.37 (0.08)	–48.32 (0.11)	–48.24 (0.09)	–48.65 (0.06)
–*T*Δ*S*	29.36 (0.39)	28.15 (0.41)	31.20 (0.46)	28.94 (0.43)
Δ*G*_bind_	–23.00 (0.40)	–20.17 (0.42)	–17.03 (0.46)	–19.71 (0.44)

a Δ*G*_elec(tot)_ = Δ*E*_elec_ + Δ*G*_PB_.

In particular, the van der Waals contribution is more pronounced compared to the electrostatic contribution in the HOX-1 complexes with H-Styr (Δ*E*_vdW_ = −80.29 kcal mol^–1^, Δ*E*_elec_ = −18.94 kcal mol^–1^, respectively), a trend that is reversed within all other derivatives considered. The examination of the electrostatic contribution to solvation (Δ*G*_PB_) reveals positive total electrostatic contributions (Δ*G*_elec(tot)_) across all complexes, indicating that electrostatic forces tend to hinder the binding of these derivatives to HOX-1, albeit to a lesser extent in the heme complex. Hence, the formation of HOX-1 complexes with these derivatives is primarily governed by the interplay between van der Waals interactions and the nonpolar contribution to solvation. The requirement for binding between iron–metalloporphyrins and HOX-1 protein to activate it is evident.^20^ The insights gleaned from MD and molecular docking simulations aid in comprehending the observed bilirubin outcomes. The binding affinities of different compounds with HOX-1 elucidate the elevated intracellular bilirubin levels. Nonetheless, the overall impact of these tested compounds on protein expression and binding affinity will ultimately determine the outcome of the HOX-1 activity.

## Conclusions

In pursuit of broader biomedical applications, particularly in breast cancer treatment, it is essential to regulate intracellular hemin levels to manage its induction of ROS generation while stimulating HOX-1 activity. This study assessed how hemin and its derivatives affect HOX-1 expression and activity, measured by bilirubin generation, as well as their impact on mitochondrial functions and ROS generation in MDA-MB-231 cells. Hemin exhibited the most significant inducing effect for HOX-1 expression, followed by its derivatives, although variations were noted in enzyme activity. H-Tyros displayed the highest inducing effects for enzyme activity, generating bilirubin, followed by hemin, whereas H-Styr showed the lowest inducing effects. Additionally, these changes were accompanied by a decrease in the expression of procaspase-3 and cleaved caspase-3, along with increased cleavage of PARP-1, which serve as markers for the extrinsic death receptor pathway of cell apoptosis. Furthermore, both hemin and H-Tyros had similar effects on mitochondrial respiration with a relative decline observed in H-Styr-treated cells. These effects were accompanied by hemin’s pro-oxidant effects, surpassing those of H-Tyros and H-Styr, leading to increased intracellular ROS generation and luminescence in the H_2_O_2_/luminol reaction. Nevertheless, the varying ROS levels in response to different compounds did not correlate with similar procaspase-3 expression. Notably, the caspase-mediated changes in mitochondrial functions under the current treatments will be examined in detail in future studies. Subsequently, a molecular docking study followed by extensive molecular dynamics simulations elucidated the interactions among the HOX-1 protein, heme, and the tested compounds. While the complexation of HOX-1 with metalloporphyrins did not induce significant structural effects, the bonding nature varied among compounds. Hydrogen bonding and hydrophobic interactions dominated the interactions of heme, hemin, and H-Tyros with the HOX-1 protein, whereas H-Styr’s complexation lacked these hydrogen bonding effects. Moreover, van der Waals and electrostatic forces mediated the complexation of HOX-1 with all compounds except H-Styr, where only van der Waals forces governed its complexation. Understanding of these interactions, influenced by metalloporphyrin characteristics, offers insights into HOX-1 regulation and ROS generation, potentially guiding the development of breast cancer therapies targeting oxidative stress. Hence, by modifying hemin’s structure and magnetic and electronic properties, its inducing effects on HOX-1 expression and activity can be tailored alongside intracellular ROS generation, thus modulating corresponding apoptosis and ferroptosis pathways.

## Experimental Section

### Materials

MDA-MB-231 cells (HTB-26) were purchased from the American Type Culture Collection. Hemin, anhydrous dimethyl sulfoxide (DMSO), styrene, tyrosine, dichloro[1,3-bis(2,4,6-trimethylphenyl)-2-imidazolidinylidene](3-phenyl-1*H*-inden-1-ylidene)(tricyclohexylphosphine)ruthenium(II) (Grubbs Catalyst M202), ethyl-3-(3′-dimethylaminopropyl)carbodiimide·HCl (EDC), silica gel (60 *A*°, 40–63 μm), thin-layer chromatography (TLC) silica gel sheets (60 *A*°, 10–12 μm), sephadex G-15, H_2_O_2_ (30%), *tert*-butyl hydroperoxide solution (tBuOOH), RPMI-1640 medium, Dulbecco’s Modified Eagle Medium (DMEM), l-glutamine, penicillin/streptomycin, FBS, sodium dihydrogen phosphate dihydrate (NaH_2_PO_4_·2H_2_O), sodium phosphate dibasic dihydrate, (Na_2_HPO_4_·2H_2_O) phosphate-buffered saline (PBS), bilirubin assay kit (MAK126), and white opaque 96-well microplates were all obtained from Sigma-Aldrich. Methanol (CH_3_OH), dichloromethane (CH_2_Cl_2_), ethyl acetate, tetrahydrofuran (THF), petroleum ether, HPLC grade water Cell-Quant alamarBlue Cell Viability Reagent, Pierce Bicinchoninic Acid Assay Protein Assay Kit, and Thermo Scientific Luminol and SuperSignal West Pico PLUS Chemiluminescent Substrate were purchased from Fisher Scientific. *N*-Hydroxysuccinimide (NHS) was from Carbosynth. The DCFDA/H2DCFDA–Cellular ROS Assay Kit (ab113851) was purchased from Abcam. The two-well silicone inserts were sourced from Ibidi, and the transwell insert with 8.0 μm pores was obtained from Cruinn Diagnostics.

### General Procedures for the Synthesis of Hemin-diester (**2**) and H-Styr (**3**)

The synthesis was previously reported,^36,37^ and started with hemin esterification,^80^ followed by cross-metathesis reaction.^81^ First, hemin diester was synthesized by hemin dissolving in a solvent mixture of CH_3_OH and H_2_SO_4_, and stirring for 20 min at room temperature, protected from light. The reaction was then quenched with ethyl acetate and H_2_O, followed by the extraction of the final products with ethyl acetate and chloroform. The resulting reddish colored solutions were dried with anhydrous sodium sulfate, filtered, and the solvent removed in vacuo. The hemin-diester was separated by using silica gel chromatography with a petroleum ether:CH_2_Cl_2_:CH_3_OH solvent mixture (1:1:0.3). For hemin–styrene conjugation, cross-metathesis reaction was performed by dissolving hemin diester in freshly distilled THF in a nitrogen-atmosphere flask fitted with a condenser at room temperature. Styrene was then injected, followed by Grubbs Catalyst, and the mixture was refluxed at 67 °C under nitrogen for 1 h. The solvent was then removed in vacuo, and the residues were purified by using a silica gel column with CH_2_Cl_2_:CH_3_OH (100:0.5), giving a final reddish-brown powder.

### General Procedures for the Synthesis of Hemin-*N*-oxysuccinimide Ester (**4**) and H-Tyrosine (**5**)

We previously reported the synthesis procedures.,^37^ with modification from the steps of Okorochenkov et al.^82^ For hemin activation, it was dissolved in DMSO under a nitrogen atmosphere and stirred for one h at room temperature in the dark. EDC and NHS were then added, and the mixture was stirred overnight under nitrogen. The product was precipitated by adding the solution to saturated NaCl, followed by diethyl ether and vacuum filtration. The dark brown hemin-*N*-oxysuccinimide ester precipitate was washed multiple times with ether and double-distilled water and then vacuum-dried. For synthesis of hemin–tyrosine conjugate, the dried hemin-*N*-oxysuccinimide ester was dissolved in DMSO under a nitrogen atmosphere and stirred for 30 min at room temperature in the dark, followed by injection of tyrosine and triethylamine.HCl. The mixture was stirred for 24 h under the same conditions, and the final product was precipitated as explained for hemin-NHS precipitation. The final product solid was purified using a Sephadex G-15 column and methanol (100%) as the mobile phase, which was finally removed in vacuo, yielding a dark brown powder.

The purity of all compounds was assessed by HPLC, elemental analysis, ^1^H NMR spectroscopy, and mass spectroscopy. The HPLC chromatograms of hemin, H-Styr, hemin–NHS conjugate, and H-Tyros are shown in SI, Figure S12A–D. The two peaks observed in the case of hemin-NHS and H-Tyros may indicate partial demetalation reactions during the elution. The purity of all compounds was ≥95% by HPLC analysis.

### Cell migration study

. The migration of MDA-MB-231 cells was assessed using transwell and scratch assays as described before.^22^ For the transwell assay, cells were incubated overnight in serum-free RPMI, trypsinized, and counted, and 80 × 10^3^ cells were seeded onto the upper chamber of each transwell insert. Compounds were added to the bottom well in FBS-containing medium at final concentrations of 4 or 8 μM. After 24 h, migrated cells on the lower surface of the membrane were fixed, stained with DAPI, and imaged using an Olympus IX81 microscope. The number of migrated cells was quantified using ImageJ software.

In the wound healing assay, 50 × 10^3^ cells were seeded in each well of a 24-well plate with a silicone insert, which was removed after 24 h. The cells were then treated with different compounds diluted in phenol red-containing, serum-free RPMI. The closure of the wound was imaged hourly for 24 h using the IncuCyte system, and the wound healing rate was analyzed with ImageJ and MiToBo toolbox.^83^ The experiment was repeated twice with three samples per group, and the average measurements were recorded.

### Western Blotting and Bilirubin Quantification

First, 400 × 10^3^ MDA-MB-231 cells were cultured in RPMI-1640 medium containing FBS in T-75 flasks and incubated for 24 h at 37 °C in 5% CO_2_ to achieve approximately 80% confluency. For in vitro testing, a stock solution of all compounds was prepared by dissolving in DMSO, followed by a fresh dilution in the culture medium. The medium was replaced with FBS-free RPMI-1640 medium containing either 4 or 8 μM of hemin, H-Tyros, or H-Styr, and cells were cultured for an additional 24 h at 37 °C in 5% CO_2_. For Western blotting, cellular proteins were extracted as detailed before,^42^ quantified using Pierce Bicinchoninic acid assay and resolved using 10% sodium dodecyl sulfate-polyacrylamide gel electrophoresis (SDS-PAGE). The following antibodies were used to detect proteins of interest: mouse monoclonal anti–HOX-1 (Invitrogen, MA1–112, 1:1000) and mouse monoclonal anti−β-actin (Sigma, A5441, 1:10000), followed by horseradish peroxidase-conjugated goat antimouse antibody (Invitrogen, 31430, 1:10000). The expression of procaspase-3, cleaved caspase 3 and PARP-1 was detected using the apoptosis western Blot Cocktail (abcam, ab136812). The protein bands were finally detected using the Thermo Scientific SuperSignal West Pico PLUS Chemiluminescent Substrate and an Omega Lum G Imaging System. Blotting was performed in triplicate, with testing of two samples per group. For bilirubin quantification, cells were lysed in deionized water, and 10 μg of protein-containing extracts were used per test. The total bilirubin was quantified using the bilirubin assay kit (MAK126, Sigma) according to the manufacturer’s protocol. with absorbance measured at 530 nm after incubation at room temperature for 10 min. Total bilirubin was quantified according to the manufacturer’s protocol, Results were normalized to the absorbance of the calibrator, with a separate blank for each sample.

### ROS Detection

First, 15 ×10^3^ MDA-MB-231 cells were seeded per each well of a 96-well plate in FBS-containing RPMI 1640 medium and allowed to attach for 24 h at 37 °C in 5% CO2. The medium was then replaced with fresh 1× supplemented buffer containing 10% FBS and 25 μM DCF-DA. Following a 45 min cell incubation, the buffer was washed three times with PBS at 10 min intervals. Subsequently, the cells were treated with phenol red and FBS-free medium containing 2, 4, or 8 μM hemin, H-Tyros, or H-Styr. Changes in fluorescence corresponding to intracellular ROS were monitored using the IncuCyte S3 Automated Live-Cell Analysis System following a 24 h cell incubation period. Four positions were randomly selected per well, with three wells per group tested, and images were acquired every hour. Assessment of fluorescence corresponding to intracellular ROS was performed automatically by IncuCyte ZOOM, measuring the green object count for all cells stained green with DCF and normalizing the results to the total object count per image. These culture and assay conditions were optimized following cell culture with different concentrations of DCF-DA in the presence of increased concentrations of H_2_O_2_ and tBuOOH. For assessment of cell viability, the metabolic activity in response to different concentrations of H_2_O_2_ and tBuOOH was measured as explained before.^36,37^

### Real-Time Measurement of Mitochondrial Functions

These functions were evaluated using the Mito Stress test by the measurement of OCR of cells using XFp Extracellular Flux Analyzer (Seahorse Bioscience, Agilent Technologies, U.K) as we described before.^22^ In brief, 20 × 10^3^ MDA-MB-231 cells were seeded in XFp Analyzer Cell Culture mini plates, then left to attach overnight at 37 °C in 5% CO_2_. The cells were then washed with unbuffered XF Base Medium containing 10 mM glucose, 1 mM sodium pyruvate, and 2 mM glutamine and incubated for 45 min at 37 °C without CO_2_. Freshly diluted tested compounds were injected into the medium, initiating the test by recording the basal OCR. Each compound was injected into the wells to achieve a final concentration of 8 μM, and OCR was recorded for 60 min. Subsequently, oligomycin (Olig), cyanide 4-(trifluoromethoxy)phenylhydrazone (FCCP), and rotenone/antimycin A (Rot/AA) were sequentially injected into each well, with concurrent recording of the OCR values. Basal, ATP-linked, and reserve capacity OCR parameters were calculated from three independent experiments conducted during the Mito Stress assays.

### Chemiluminescence Measurements

The effects of different compounds on the H_2_O_2_/luminol luminescence kinetics were evaluated following dilution in both phosphate buffer and FBS-free DMEM as reported before. Luminol was initially dissolved in DMSO, and freshly diluted in phosphate buffer with fresh dilution of H_2_O_2_ in the buffer. In brief, H_2_O_2_ and luminol were injected into solutions containing hemin, H-Tyros or H-Styr in white opaque 96-well microplates for final concentrations of 50 and 1 mM, respectively. The luminescence intensity was measured instantly, followed by continuous recording over three h at two min intervals using the luminescence option in the Varioskan Flash microplate reader (Thermo ScientificTM, Finland).

### Molecular Docking Studies

The PDB structure for HOX-1 (PDB 1N45) was retrieved from the Brookhaven Protein Data Bank (www.rcsb.org/structure/1n45).^84^ The details for receptor preparation and optimization of geometry of tested compounds are in the Supporting Information. Consequently, the HOX-1 receptor and the heme, hemin, H-Tyros and H-Styr structures were prepared for molecular docking calculations using the AutoDock Vina software package^85,86^ with the AutoDock Tools 1.5.7 python libraries.^87,88^ Partial atomic charges were assigned to HOX-1 according to the Kollman United Atom scheme; for heme, hemin, and H-Tyros and H-Styr RESP charges were used. The starting geometry for hemin was obtained from the crystal structure of Koenig, 1965,^89^ optimized and modified as reported before.^36,37^ It has been previously demonstrated that the combination of AM1-BCC or RESP charges for ligands and Amber99SB charges for proteins performs statistically better than the standard combination of Gasteiger charges for ligands and proteins.^90^ In both protein and ligand structures, nonpolar hydrogens were merged to heavy atoms, while the torsion tree and the rotatable/nonrotatable bonds present were also set.^87,88^ Docking calculations were performed using the AD4 scoring function, which comprises five energetic terms: the van der Waals interaction, the hydrogen bonding interaction, the electrostatic interaction, the desolvation energy, and the torsional entropy. Grid spacing was set to 0.375 Å. The grid center was placed at the crystallographic heme’s center of mass coordinates, while the optimal grid box dimensions were calculated from the radius of gyration of each derivative (SI, Table S4).^91^ The global searching exhaustiveness was set equal to 300 and a total of 100 binding modes were retained from each calculation. SI, Table S8 shows the grid box sizes used during the molecular docking calculations.

### Molecular Dynamics Simulations

Molecular dynamics (MD) simulations were prepared and executed using Enalos Asclepios KNIME nodes (Figure S1). Initially, the produced parameters with the R.E.D. server as well as the best scoring docking conformation of each derivative were used as input to the “AsclepiosAmberSystemsPrep” node, which handles the preparation of the model protein–ligand complexes with AmberTools21.^92^ The AMBER14SB^93^ force field was assigned to the HOX-1 protein, while water solvent molecules were explicitly added by means of the TIP3P model,^94^ with a 10 Å buffer around the complexes, using truncated octahedron unit cells and applying periodic boundary conditions in each direction. Electro neutrality of the total system charge was achieved by adding Na^+^ ions. The MD simulations were performed with the AsclepiosAmberMDSimulation node utilizing the OpenMM 7.5^95^ software and consisted of the following stages: energy minimization, equilibration simulations in the canonical (NVT) and isothermal–isobaric (NPT) ensembles, followed by the production simulations and postprocessing analysis. Energy minimization was performed for 20,000 steps imposing positional restraints on the protein–ligand complex atoms with a harmonic force constant. The restraint force was gradually lifted every 5000 steps starting from 100 to 20, 2, and 0 kcal mol^–1^ Å^–2^. To conserve the Fe(III) coordination to H^25^ nitrogen, a soft harmonic force constant restraint was imposed, equal to the calculated force constant of the Fe–N bond. The energy minimized systems were then subjected to an initial equilibration stage for 1 ns with a 1 fs time step with the following protocol. Using a Langevin thermostat,^96^ each system was first heated in the NVT ensemble for 200 ps, gradually raising the temperature from 0 to 300 K in 3 ps increments while applying a harmonic force constant of 20 kcal mol^–1^ Å^–2^ to the heavy the protein–ligand complex atoms. Then, 800 ps NPT simulations commenced, using a Monte Carlo barostat^97,98^ to gradually increase the pressure from 0.1 atm to the target value (i.e., 1.0 atm), imposing the same harmonic force constants. The restraint force was gradually reduced from 20, to 2 and 0 kcal mol^–1^ Å^–2^ over a 1 ns period, followed by another 1 ns of unrestrained NPT simulations. After completion of these steps, the potential energy and density converged to the mean values. The Particle Mesh Ewald method^99^ was employed for computing long-range electrostatic interactions, utilizing a 10 Å cutoff for both electrostatic and LJ interactions. Bonds involving hydrogen atoms were constrained, allowing for a 2 fs time step throughout each of the 200 ns production simulations, which were conducted three times with initial velocities randomly assigned to ensure proper statistics.

A total of 5000 frames were saved from each trajectory and used for analysis performed with the cpptraj module of AmberTools21^92^ within the same node, which include root-mean-square deviation (RMSD), root-mean-squared fluctuations (RMSF), HB and clustering analysis. Specifically, for Cα atoms (HOX-1) and all heavy atoms (heme and hemin derivatives), the RMSD values are mass-weighted and calculated with respect to the initial system coordinates. RMSF were calculated for the heavy protein backbone atoms, with mass-weighted averages performed to obtain a single value per amino acid. For HB analysis, a distance cutoff of 3.5 Å and a donor-hydrogen-acceptor angle of 150° criterion is used. Clustering analysis is performed using the dbscan algorithm,^100^ setting the midpoints, epsilon and sieve values to 25, 3, and 10 frames, respectively. The HOX-1 active site cavity was analyzed and characterized by means of the MDpocket software.^101^

The binding free energies were evaluated with the “AsclepiosAmberMMPBGBSA” node with the Molecular Mechanics Poisson–Boltzmann Surface Area (MM-PBSA) method. The method calculates the interaction energy in the gas phase with molecular mechanics and estimates the solvation free energy by solving the Poisson–Boltzmann equation.^102,103^ The corresponding equations can be found in the Supporting Information. While absolute binding energy calculations using MM–PBSA may not consistently match experimental findings, studies have demonstrated its efficacy in predicting relative binding energies.^104^ The calculations were conducted using the Asclepios implementation of the AmberTools21 MMPBSA.py^105^ script of the respective KNIME workflow node over the last 2,500 frames of each trajectory (i.e., 7500 frames for each complex were considered). For the surface tension (γ) and the offset (β), values of 0.00720 kcal mol^–1^ Å^–2^ and 0.0000 kcal mol^–1^, respectively, were considered. The default probe radius of 1.4 Å was applied to the solvent (water) in the *SASA* calculation. The evaluation of conformational entropy contribution was conducted via normal-mode analysis, employing a 50-frame offset for each analysis, commonly recommended practice for computational efficiency considerations,^105^ given the computationally intensive nature of these calculations. Statistical uncertainties are presented as the standard errors of the mean (SEM). Per-residue contributions to the total enthalpy of each system were calculated for all complexes considered.

### Statistical Analysis

The results were analyzed using SPSS statistical software (IBM SPSS statistics 26). All data were expressed as the means ± SD. Data were analyzed using a *t* test, and the differences were considered statistically significant at (*p* < 0.05).

## Data Availability

The molecular structures and AMBER force field files used in the MD simulations are available upon request.

## Supporting Information Available

XYZ-files of optimized geometry of heme (FeC_34_N_4_H_32_O_4_) (S = 1), hemin (FeC_34_N_4_H_32_O_4_Cl) (S = 5/2), H-Tyros (FeC_52_N_6_H_50_O_8_Cl) (S = 5/2), and H-Styr (FeC_48_N_4_H_44_O_4_Cl) (S = 5/2); binding affinities of heme, hemin, H-Tyros, and H-Styr in the HOX-1 protein determined via molecular docking; per-residue MM–PBSA energy decomposition of the HOX-1 complexes with heme, hemin, H-Tyros, and H-Styr; average center of mass distances (in Å) between the Fe(III) coordinated water molecule of heme and the chloride atom of hemin, H-Tyros, and H-Styr derivatives with the A^140^, G^143^, and L^147^ HOX-1 residues; grid box sizes used during the molecular docking calculations; Enalos Asclepios KNIME workflow for the preparation and execution of MD simulations; effects of H_2_O_2_ and tBuOOH on the metabolic activity of MDA-MB-231; kinetics of the changes in intracellular ROS levels revealed by the ROS-specific indicator DCF-DA and its fluorescence in MDA-MB-231 cells; luminescence kinetics following addition of 50 mM H_2_O_2_ and 1 mM luminol in phosphate buffer to hemin-, H-Tyros-, and H-Styr-containing solutions; RMSD trajectory analyses of HOX-1 protein complexes with the heme, hemin, H-Tyros, and H-Styr derivatives; centroid structure of heme, hemin, H-Tyros, and H-Styr derivatives in complex with HOX-1; centroid structure of the HOX-1 protein complex with heme; HOX-1 protein’s radius of gyration (Rg) in complex with heme, hemin, H-Tyros, and H-Styr derivatives; solvent-accessible surface area (SASA) of the HOX-1 protein in complex with heme, hemin, H-Tyros, and H-Styr derivatives; calculated average pocket volume of the HOX-1 protein with its complexes with heme, hemin, H-Tyros, and H-Styr derivatives; average COM distances between the Fe(III) coordinated water and chlorine atom of heme and hemin; comparison of the HPLC chromatograms of hemin, H-styr, hemin-NHS conjugate and H-Tyros (PDF)
Molecular formula strings (CSV)
Time-dependent migration of untreated MDA-MB-231 cells using a scratch assay, and the accompanying changes in the gap area (AVI)
Time-dependent migration of MDA-MB-231 cells, treated with 4 μM hemin, using a scratch assay, and the accompanying changes in the gap area (AVI)
Time-dependent migration of MDA-MB-231 cells, treated with 8 μM hemin, using a scratch assay, and the accompanying changes in the gap area (AVI)
Time-dependent migration of MDA-MB-231 cells, treated with 4 μM H-Styr, using a scratch assay, and the accompanying changes in the gap area (AVI)
Time-dependent migration of MDA-MB-231 cells, treated with 8 μM H-Styr, using a scratch assay, and the accompanying changes in the gap area (AVI)
Time-dependent migration of MDA-MB-231 cells, treated with 4 μM H-Tyros, using a scratch assay, and the accompanying changes in the gap area (AVI)
Time-dependent migration of MDA-MB-231 cells, treated with 8 μM H-Tyros, using a scratch assay, and the accompanying changes in the gap area (AVI)
Time-dependent changes in levels of intracellular ROS, in DCF-DA-labeled MDA-MB-231 cells, without any treatment (MP4)
Time-dependent changes in levels of intracellular ROS, in unlabeled MDA-MB-231 cells, without any treatment (MP4)
Time-dependent changes in levels of intracellular ROS, in DCF-DA-labeled MDA-MB-231 cells, treated with 2 μM hemin (MP4)
Time-dependent changes in levels of intracellular ROS, in DCF-DA-labeled MDA-MB-231 cells, treated with 4 μM hemin (MP4)
Time-dependent changes in levels of intracellular ROS, in DCF-DA-labeled MDA-MB-231 cells, treated with 8 μM hemin (MP4)
Time-dependent changes in levels of intracellular ROS, in DCF-DA-labeled MDA-MB-231 cells, treated with 2 μM H-Tyros (MP4)
Time-dependent changes in levels of intracellular ROS, in DCF-DA-labeled MDA-MB-231 cells, treated with 4 μM H-Tyros (MP4)
Time-dependent changes in levels of intracellular ROS, in DCF-DA-labeled MDA-MB-231 cells, treated with 8 μM H-Tyros (MP4)
Time-dependent changes in levels of intracellular ROS, in DCF-DA-labeled MDA-MB-231 cells, treated with 2 μM H-Styr (MP4)
Time-dependent changes in levels of intracellular ROS, in DCF-DA-labeled MDA-MB-231 cells, treated with 4 μM H-Styr (MP4)
Time-dependent changes in levels of intracellular ROS, in DCF-DA-labeled MDA-MB-231 cells, treated with 8 μM H-Styr (MP4)

## Supplementary Material

jm4c00989_si_002.csv

jm4c00989_si_003.avi

jm4c00989_si_004.avi

jm4c00989_si_005.avi

jm4c00989_si_006.avi

jm4c00989_si_007.avi

jm4c00989_si_008.avi

jm4c00989_si_009.avi

## Abbreviations Used

CL: chemiluminescence
CO: carbon monoxide
COM: the center of mass
DCF-DA: 2′,7’-dichlorofluorescein diacetate
DMEM: Dulbecco’s Modified Eagle Medium
DMSO: dimethyl sulfoxide
EDC: 1-ethyl-3-(3′-dimethylaminopropyl)carbodiimide·HCl
FCCP: cyanide 4-(trifluoromethoxy)phenylhydrazone
FBS: fetal bovine serum
Fe(II)-PPIX: heme
PPIX-Fe(III): ferryl heme (hemin)
HB: hydrogen bond
HO^●^: hydroxyl radical
HOO^●^: hydroperoxyl radical
H_2_O_2_: hydrogen peroxide
HOX-1: heme oxygenase-1
H-Tyros: hemin-tyrosine conjugate
H-Styr: hemin–styrene conjugate
MM–PBSA: mechanics Poisson–Boltzmann surface area
NHS: *N*-hydroxysuccinimide
Olig: oligomycin
PARP-1: poly[ADP-ribose] polymerase 1
Rg: radius of gyration
ROS: reactive oxygen species
Rot/AA: rotenone/antimycin A
RMSD: root-mean-square deviation
RMSF: root-mean-square fluctuations
SASA: solvent-accessible surface area
tBuOOH: *tert*-butyl hydroperoxide
