# Supplementary material for: Examining Hemin and its Derivatives: Induction of Heme-Oxygenase-1 Activity and Oxidative Stress in Breast Cancer Cells through Collaborative Experimental Analysis and Molecular Dynamics Simulations
Source: J Med Chem. 2024 Aug 19;67(17):15411–27. doi: 10.1021/acs.jmedchem.4c00989 (PMC11403666; doi:10.1021/acs.jmedchem.4c00989)
Supplement: Supplementary file 1 — jm4c00989_si_001.pdf [file jm4c00989_si_001.pdf]

Supporting Information

Examining Hemin and its Derivatives: Induction of Heme-Oxygenase-1 Activity and Oxidative Stress in Breast Cancer Cells through Collaborative Experimental Analysis and Molecular Dynamics Simulations

Amir M. Alsharabasy,[1*] Panagiotis I. Lagarias,[2] Konstantinos D. Papavasileiou,[2,3,4] Antreas Afantitis,[2,3,4] Pau Farràs,[1,5] Sharon Glynn,[1,6] Abhay Pandit[1*]

[1]CÚRAM, SFI Research Centre for Medical Devices, University of Galway, Ireland. Postal code: H91 W2TY.
[2]Department of ChemoInformatics, Novamechanics Ltd., Nicosia, Cyprus. Postal code: 1070
[3]Department of Chemoinformatics, Novamechanics MIKE, Piraeus, Greece. Postal code: 18545
[4]Division of Data Driven Innovation, Entelos Institute, Larnaca, Cyprus. Postal code: 6059
[5]School of Biological and Chemical Sciences, Ryan Institute, University of Galway, Ireland. Postal code: H91 TK33.
[6]Discipline of Pathology, Lambe Institute for Translational Research, School of Medicine, University of Galway, Ireland. Postal code: H91 YR71.

*AP: E-mail: Abhay.pandit@universityofgalway.ie
*AMA: E-mail: Amir.abdo@universityofgalway.ie

TABLE OF CONTENT

List of table captions/legends

Supplementary Table S1. XYZ-files of optimized geometry of heme ($FeC_{34}N_4H_{32}O_4$) (B3LYP, GenECP, S = 1). The basis sets: LANL2DZ for Fe atom; 6-31+G(d,p) for H atom**,** 6-31+G(d,p) for C, O and N atoms………........**S-6**
Supplementary Table S2. XYZ-files of optimized geometry of hemin ($FeC_{34}N_4H_{32}O_4Cl$) (B3LYP, GenECP, S = 5/2). The basis sets: LANL2DZ for Fe atom; 6-31+G(d,p) for H atom**,** 6-31+G(d,p) for C, O, N and Cl atoms......**S-8**
Supplementary Table S3. XYZ-files of optimized geometry of H-Tyros ($FeC_{52}N_6H_{50}O_8Cl$) (B3LYP, GenECP, S = 5/2). The basis sets: LANL2DZ for Fe atom; 6-31+G(d,p) for H atom**,** 6-31+G(d,p) for C, O, N and Cl atoms….**S-10**
Supplementary Table S4. XYZ-files of optimized geometry of H-Styr ($FeC_{48}N_4H_{44}O_4Cl$) (B3LYP, GenECP, S = 5/2). The basis sets: LANL2DZ for Fe atom; 6-31+G(d,p) for H atom**,** 6-31+G(d,p) for C, O, N and Cl atoms... **S-14**
Supplementary Table S5. Binding affinities (in kcal mol^{-1}) of heme, hemin, H-Tyros, and H-Styr in the HOX-1 protein determined via molecular docking using the Autodock Vina software…………………………………. **S-22**

Supplementary Table S6. Per-residue MM–PBSA energy decomposition of the HOX-1 complexes with heme, hemin, H-Tyros and H-Styr. Only the most favorable residues are shown. Uncertainties denote standard error of the mean (units in kcal mol^{-1}) and are included in parentheses...…………………………… …………………..... **S-24**

Supplementary Table S7. Average center of mass distances (in Å) between the Fe(III) coordinated water molecule of heme and the chloride atom of hemin, H-Tyros, and H-Styr derivatives with the A^{140}, G^{143}, and L^{147} HOX-1 residues, calculated from three replicate trajectories. Uncertainties are indicated in parentheses………..…….. **S-27**

Supplementary Table S8. Grid box sizes used during the molecular docking calculations.………… ……..... **S-31**

List of figure captions/legends

Supplementary Figure S1. The Enalos Asclepios KNIME workflow for the preparation and execution of MD simulations performed in this study..…………………………………………………………………….. ……..**S-6**

Supplementary Figure S2. Effects of H_2O_2 and tBuOOH on the metabolic activity of MDA-MB-231 after 24 hours of culture **(a)** and the levels of ROS detected by DCF-fluorescence after 12 and 24 hours of culture **(b)**. The cells were treated either directly with different concentrations of H_2O_2 and tBuOOH for assessment of cell viability, or with prior treatment with 10, 25 or 40 μM DCF-DA for monitoring the changes in intracellular ROS levels. Results are presented as mean ± S.D, n = 3. *, $P < 0.05$ versus the control group (untreated cells) in case of H_2O_2-treated cells; #, $P < 0.05$ versus the control group (untreated cells) in case of H_2O_2-treated cells using a two-tailed unpaired student t-test**…………………………………………………………………………………………………. S-18**

Supplementary Figure S3. (a) Kinetics of the changes in intracellular ROS levels revealed by the ROS-specific indicator DCF-DA and its fluorescence in MDA-MB-231 cells in response to 2, 4 and 8 μM hemin in FBS-free and FBS-containing media. **(b)** The change in fluorescence in case of FBS-containing medium only with different treatments. The cells were treated with DCF-DA for 45 min, and then photographed after adding the different treatments using the real-time Incucyte® imaging system (phase contrast and green fluorescence signals). Results are presented as mean fluorescence intensity, n = 3……..…………………………………..…………………**S-19**

Supplementary Figure S4. The luminescence kinetics following addition of 50 mM H_2O_2 and 1 mM luminol in phosphate buffer (50 mM, pH 7.4) to hemin-**(a)**, H-Tyros-**(b)**, and H-Styr **(c)**-containing phosphate buffer **(solid curves)** or FBS-free DMEM **(dotted curves)** at the concentrations 4 **(red curves)** and 8 μM **(blue curves).** Results are presented as mean luminescence intensity values, n = 3..…………………….……………………….. ….**S-21**

Supplementary Figure S5. RMSD trajectory analyses of HOX-1 protein complexes with the **(a)** heme, **(b)** hemin, **(c)** H-Tyros and **(d)** H-Styr derivatives. ………………………………………………………..……..……..**S-23**

Supplementary Figure S6. Centroid structure of **(a)** heme, **(b)** hemin, **(c)** H-Tyros and **(d)** H-Styr derivatives in complex with HOX-1, determined from clustering conformational analysis of the MD simulation trajectories. Figure was prepared with ChimeraX.[24-26] Hydrogen bonds (red) and close contacts (green) with HOX-1 residues as also illustrated as dashed lines..…………………………………………………………..……………………**S-24**

Supplementary Figure S7. The centroid structure of the HOX-1 protein complex with heme, showcasing the water-mediated hydrogen bonding interaction involving the Fe(III) coordinated water and G^{139} and G^{143} residues. Residues H^{25} and E^{29}, which are hydrogen bonded for the majority of the simulation time, are also illustrated.………….**S-27**
Supplementary Figure S8. HOX-1 protein's radius of gyration (Rg) in complex with **(a)** heme, **(b)** hemin, **(c)** H-Tyros, and **(d)** H-Styr derivatives. Solid lines depict the average Rg value, while shaded regions indicate the respective standard deviation from three independent simulations. Average values are equal to **(a)** 17.25 ± 0.07, **(b)** 17.28 ± 0.09, **(c)** 17.28 ± 0.08 and **(d)** 17.26 ± 0.07 Å, respectively...………… …………..……..………..……..**S-28**
Supplementary Figure S9. The solvent-accessible surface area (SASA) of the HOX-1 protein in complex with **(a)** heme, **(b)** hemin, **(c)** H-Tyros, and **(d)** H-Styr derivatives. Solid lines indicate the average SASA value, while shaded regions represent the respective standard deviation from three independent simulations. Average values are equal to a) 10.44 ± 0.18, b) 10.46 ± 0.21, c) 10.48 ± 0.19 and d) 10.46 ± 0.20 $\times 10^3$Å^2, respectively.……..………**S-29**
Supplementary Figure S10. Calculated average pocket volume of the HOX-1 protein including the standard deviation in its complexes with heme, hemin, H-Tyros, and H-Styr derivatives, measured over the last 100 ns of all trajectories using the MDpocket program.[27] Average values are equal to 1399.9 ± 133.1, 1428.5 ± 142.4, 1532.5 ± 135.9 and 1416.8 ± 155.5 Å^3, respectively...……………………………. ……….………………………..…..**S-30**
Supplementary Figure S11. The average COM distances between the Fe(III) coordinated water and chlorine atom of heme and hemin, respectively, and D^{140}, G^{143} and L^{147} residues in the heme, hemin, H-Tyros, and H-Styr complexes with HOX-1……………………………………….………..……………………………..…..**S-30**
Supplementary Figure S12. Comparison of the HPLC chromatograms of **(a)** hemin, **(b)** H-styr, **(c)** hemin-NHS conjugate and **(d)** H-Tyros...……………………………………………..……………….………..……..**S-31**
Supplementary Video S1. The time-dependent migration of untreated MDA-MB-231 cells using a scratch assay, and the accompanying changes in the gap area. Scale bar: 800 µm. ...…………………………. ……..……..**S-18**
Supplementary Video S2. The time-dependent migration of MDA-MB-231 cells, treated with 4 µM hemin, using a scratch assay, and the accompanying changes in the gap area. Scale bar: 800 µm…………………. ……..……..**S-18**
Supplementary Video S3. The time-dependent migration of MDA-MB-231 cells, treated with 8 µM hemin, using a scratch assay, and the accompanying changes in the gap area. Scale bar: 800 µm. .... ………. ……..……..**S-18**
Supplementary Video S4. The time-dependent migration of MDA-MB-231 cells, treated with 4 µM H-Styr, using a scratch assay, and the accompanying changes in the gap area. Scale bar: 800 µm. .... ………. ……..……..**S-18**
Supplementary Video S5. The time-dependent migration of MDA-MB-231 cells, treated with 8 µM H-Styr, using a scratch assay, and the accompanying changes in the gap area. Scale bar: 800 µm. .... ………. ……..……..**S-18**
Supplementary Video S6. The time-dependent migration of MDA-MB-231 cells, treated with 4 µM H-Tyros, using a scratch assay, and the accompanying changes in the gap area. Scale bar: 800 µm. .... ….…. ……..……..**S-18**
Supplementary Video S7. The time-dependent migration of MDA-MB-231 cells, treated with 8 µM H-Tyros, using a scratch assay, and the accompanying changes in the gap area. Scale bar: 800 µm. .... …. ……..…..……..**S-18**
Supplementary Video S8. The time-dependent changes in levels of intracellular ROS, in DCF-DA-labelled MDA-MB-231 cells, without any treatment. Scale bar: 400 µm. .... ………. ………………………..……..……..**S-21**

Supplementary Video S9. The time-dependent changes in levels of intracellular ROS, in unlabelled MDA-MB-231 cells, without any treatment. Scale bar: 400 µm. ……………………..…………………………..…..……..**S-21**

Supplementary Video S10. The time-dependent changes in levels of intracellular ROS, in DCF-DA-labelled MDA-MB-231 cells, treated with 2 µM hemin. Scale bar: 400 µm. ……………………………………..…..……..**S-21**

Supplementary Video S11. The time-dependent changes in levels of intracellular ROS, in DCF-DA-labelled MDA-MB-231 cells, treated with 4 µM hemin. Scale bar: 400 µm. ……………………………………..…..……..**S-22**

Supplementary Video S12. The time-dependent changes in levels of intracellular ROS, in DCF-DA-labelled MDA-MB-231 cells, treated with 8 µM hemin. Scale bar: 400 µm. ……………………………………..…..……..**S-22**

Supplementary Video S13. The time-dependent changes in levels of intracellular ROS, in DCF-DA-labelled MDA-MB-231 cells, treated with 2 µM H-Tyros. Scale bar: 400 µm. …………………………………….…..……..**S-22**

Supplementary Video S14. The time-dependent changes in levels of intracellular ROS, in DCF-DA-labelled MDA-MB-231 cells, treated with 4 µM H-Tyros. Scale bar: 400 µm. …………..………………………..…..……..**S-22**

Supplementary Video S15. The time-dependent changes in levels of intracellular ROS, in DCF-DA-labelled MDA-MB-231 cells, treated with 8 µM H-Tyros. Scale bar: 400 µm. …………………………………….…..……..**S-22**

Supplementary Video S16. The time-dependent changes in levels of intracellular ROS, in DCF-DA-labelled MDA-MB-231 cells, treated with 2 µM H-Styr. Scale bar: 400 µm. …………………………………….…..……..**S-22**

Supplementary Video S17. The time-dependent changes in levels of intracellular ROS, in DCF-DA-labelled MDA-MB-231 cells, treated with 4 µM H-Styr. Scale bar: 400 µm. …………………………………….…..……..**S-22**

Supplementary Video S18. The time-dependent changes in levels of intracellular ROS, in DCF-DA-labelled MDA-MB-231 cells, treated with 8 µM H-Styr. Scale bar: 400 µm. …………………………………….…..……..**S-22**

EXPERIMENTAL SECTION

Molecular docking studies

The PDB structure for heme-oxygenase-1 (ID: 1N45) was retrieved from the Brookhaven Protein Data Bank (www.rcsb.org/structure/1n45).[1] The A chain of the receptor was prepared by removing all heteroatoms, replacing

non-standard residues, adding heavy atoms and adjusting hydrogen atoms at neutral pH by means of PDBFixer[2] and the AmberTools21[3] *pdb4amber* utility implemented in the Enalos Asclepios KNIME pipeline (**Figure S1**).[4,5] Atomic charge derivation, atom typing, construction of force field libraries, and generation of force field parameters for the compounds under investigation were performed utilizing the PyRED program accessible through the R.E.D. Server Development website.[6-9] This was achieved by partitioning the B3LYP/GENECP (LANL2DZ for Fe, 6-311+g(d,p) for C, H, O and Cl atoms, 6-31+g(d,p) for H atoms) optimized structures of heme, hemin chloride, H-Tyros, and H-Styr into constituent fragments. These fragments represent distinct input molecules or basic building units, including heme and hemin chloride monomers, along with a list of six organic compounds: ethane, propene, pentanoate, tyrosine, styrene, and methyl hexanoate. The porphyrin ring of the monomeric heme/hemin chloride derivatives features methyl capping groups instead of the typical vinyl and propionic acid groups, a containing covalently ferric iron to the four coordinated heminic nitrogen atoms in heme and Cl in hemin chroride, bearing a non-integer partial charge. For the heme and hemin chloride building blocks the high spin state (S = 5/2) of Fe(III) was considered in the electrostatic potentials (ESP) calculations using the Gaussian 2009 program (version D.01) at the B3LYP/6-31G* level of theory[10-12] for each optimized geometry. Molecular orientations were automatically selected using a rigid-body reorientation algorithm[6] and calculation of RESP atomic charges was performed with the standard two-stage fitting procedure,[8] imposing the appropriate intra-molecular charge constraints for the capping groups. The PyRED program utilized an internal dictionary of atom types and a database of force field parameters aligned with the Amber99 force field to determine atom types and force field parameters for the input molecules,[13] with missing parameters assigned by analogy to pre-existing ones. The determination of force field parameters for the covalently bound ferric ion proceeded as follows: i) bond and angle force field constants were derived from the Hessian matrix, utilizing the Gaussian formatted checkpoint file obtained from frequency computation according to Seminario's method[14] via the VFFDT software.[15] ii) The energy barrier for dihedral force field parameters was set to zero, while iii) improper force field parameters were omitted from consideration. Finally, van der Waals parameters for the Fe(III) atom were sourced from the Amber 12 force field. The generated force field parameters are provided in the Supporting Information.

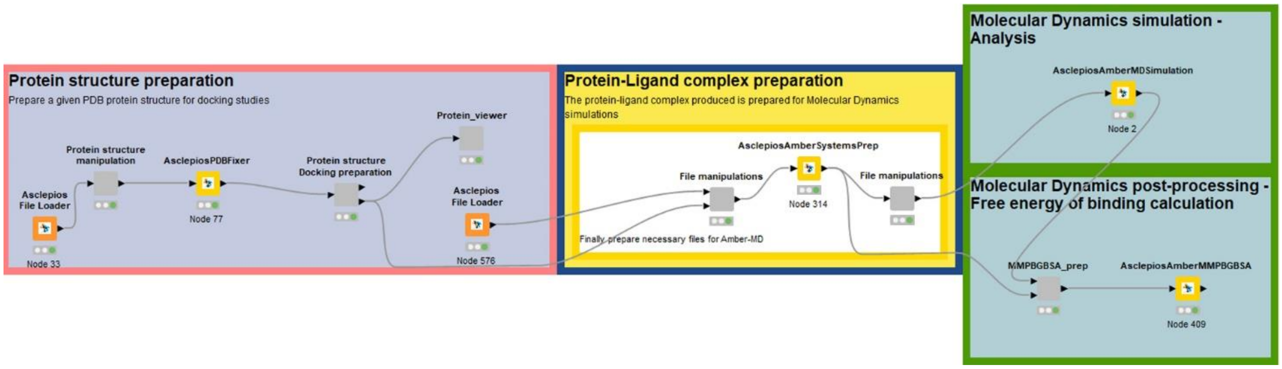

Figure S1. The Enalos Asclepios KNIME workflow for the preparation and execution of MD simulations performed in this study.

RESULTS and DISCUSSION

Table S1. XYZ-files of optimized geometry of heme ($FeC_{34}N_4H_{32}O_4$) (B3LYP, GenECP, S = 1). The basis sets: LANL2DZ for Fe atom; 6-31+G(d,p) for H atom**,** 6-31+G(d,p) for C, O and N atoms.

	X	Y	Z
**Fe**	-1.10184	-0.11013	-0.22391
**O**	5.32577	1.44151	0.13002
**O**	5.2543	1.95898	2.27207
**O**	7.03942	-2.94236	0.41808
**O**	6.98911	-0.72866	0.30252
**N**	-2.24871	-1.7422	-0.04382
**N**	0.5042	-1.26514	-0.49144
**N**	0.0547	1.51493	-0.32624
**N**	-2.7139	1.04875	-0.00991
**C**	-3.59276	-1.79725	0.25475
**C**	2.24229	0.45887	-0.61748
**C**	1.42406	1.57006	-0.46986
**C**	1.89421	2.9459	-0.46569
**C**	0.7813	3.73174	-0.31808
**C**	-0.35102	2.82897	-0.24413
**C**	-1.66832	3.25228	-0.1485
**C**	-2.77897	2.42234	-0.08515
**C**	-4.1579	2.88383	0.0226
**C**	-4.9226	1.7566	0.22178
**C**	-4.01488	0.63343	0.18587
**C**	-4.04741	-3.1799	0.35094
**C**	-4.41906	-0.68665	0.33342
**C**	-2.86726	-5.45744	-0.01065
**C**	2.30071	-4.55814	-0.87175
**C**	4.19843	-1.94017	-0.86436
**C**	4.86333	-1.91258	0.5411
**C**	3.31871	3.41353	-0.61327
**C**	-2.95772	-3.9616	0.04128
**C**	4.13499	3.46394	0.70198
**C**	0.68566	5.2271	-0.25125

**C**	-6.40875	1.6567	0.39402
**C**	-1.85281	-3.05679	-0.17699
**C**	-0.5558	-3.47351	-0.44721
**C**	0.54494	-2.64137	-0.57225
**C**	1.90294	-3.11646	-0.7593
**C**	2.69938	-2.00327	-0.77962
**C**	1.81438	-0.86114	-0.62243
**C**	-5.38117	-3.66858	0.69902
**C**	-6.25567	-3.12002	1.55725
**C**	4.92767	2.20697	0.99767
**C**	6.38026	-1.91897	0.43345
**C**	-4.65883	4.25547	-0.06085
**C**	-4.18003	5.25756	-0.81506
**H**	4.57857	-2.81227	-1.40755
**H**	3.3031	0.63822	-0.73648
**H**	-1.8443	4.31963	-0.10514
**H**	-5.4751	-0.86493	0.49153
**H**	2.12878	-5.10087	0.06685
**H**	3.36115	-4.66078	-1.11928
**H**	1.72726	-5.07624	-1.65006
**H**	0.32877	5.56547	0.72998
**H**	1.65584	5.70034	-0.4261
**H**	-0.01413	5.62123	-0.99844
**H**	4.518	-1.05819	-1.43078
**H**	4.56978	-2.80689	1.09643
**H**	3.86341	2.79289	-1.3318
**H**	3.30914	4.42406	-1.03152
**H**	4.88632	4.26361	0.63582
**H**	3.49257	3.71485	1.55268
**H**	-0.38547	-4.54104	-0.53459
**H**	-5.6656	-4.60822	0.22495
**H**	-7.21969	-3.58613	1.74002
**H**	-6.03882	-2.21648	2.11924
**H**	-4.64848	6.23748	-0.79711
**H**	-5.54713	4.45865	0.53771
**H**	-3.33508	5.13551	-1.48613

**H**	4.87926	2.63037	2.86973
**H**	6.34924	0.02647	0.34011
**H**	-2.41166	-5.80151	-0.9469
**H**	-2.25321	-5.85401	0.8086
**H**	-3.854	-5.92141	0.06935
**H**	4.51669	-1.03579	1.09701
**H**	-6.86684	2.6437	0.50023
**H**	-6.67378	1.06864	1.28098
**H**	-6.88337	1.16792	-0.46689

Table S2. XYZ-files of optimized geometry of hemin ($FeC_{34}N_4H_{32}O_4Cl$) (B3LYP, GenECP, S = 5/2). The basis sets: LANL2DZ for Fe atom; 6-31+G(d,p) for H atom**,** 6-31+G(d,p) for C, O, N and Cl atoms.

	**X**	**Y**	**Z**
**Fe**	0.910965	0.114876	-0.20684
**Cl**	0.302076	-0.154	2.081383
**O**	-4.97005	-1.68338	1.493195
**O**	-3.0561	-0.94859	2.295053
**O**	-7.35123	1.863872	-0.61931
**O**	-7.00112	0.050934	0.613495
**N**	1.903204	1.84871	-0.13982
**N**	-0.72096	1.111751	-0.81418
**N**	0.010401	-1.59097	-0.69159
**N**	2.679311	-0.84458	-0.28305
**C**	3.192433	2.019412	0.310258
**C**	-2.28449	-0.76196	-0.80831
**C**	-1.35028	-1.76486	-0.61655
**C**	-1.66563	-3.15349	-0.38135
**C**	-0.4787	-3.84039	-0.43296
**C**	0.555466	-2.85044	-0.61459
**C**	1.908987	-3.1301	-0.65245
**C**	2.905426	-2.17555	-0.54392
**C**	4.324731	-2.46842	-0.47621
**C**	4.940069	-1.30463	-0.07275
**C**	3.907234	-0.30768	0.027802
**C**	3.4768	3.429684	0.500238
**C**	4.136813	1.013114	0.376025

**C**	2.13872	5.589998	0.025803
**C**	-2.77535	4.176713	-1.51816
**C**	-4.4285	1.407497	-1.33182
**C**	-5.08927	1.473314	0.069078
**C**	-2.99845	-3.65979	0.081582
**C**	2.364374	4.110361	0.061784
**C**	-3.24049	-3.31523	1.585594
**C**	-0.23503	-5.3083	-0.26236
**C**	6.398202	-1.06037	0.162864
**C**	1.391861	3.115608	-0.30811
**C**	0.113556	3.4078	-0.75353
**C**	-0.87516	2.468718	-0.97413
**C**	-2.25167	2.794771	-1.27645
**C**	-2.94672	1.616189	-1.23821
**C**	-1.97842	0.577448	-0.9597
**C**	4.700992	4.022431	1.033138
**C**	5.455768	3.532225	2.023603
**C**	4.980722	-3.74088	-0.76142
**C**	4.624225	-4.63484	-1.69208
**C**	-3.81489	-1.92201	1.785617
**C**	-6.57954	1.163304	-0.0131
**H**	-4.88827	2.163272	-1.97093
**H**	-3.32603	-1.04606	-0.84941
**H**	2.213155	-4.16584	-0.68412
**H**	5.143996	1.293933	0.644611
**H**	-2.69614	4.798735	-0.62052
**H**	-3.82621	4.153643	-1.80823
**H**	-2.22184	4.681095	-2.31575
**H**	0.290226	-5.52362	0.673631
**H**	-1.17022	-5.8694	-0.25559
**H**	0.377941	-5.70613	-1.07642
**H**	-4.6487	0.441879	-1.7955
**H**	-3.82765	-3.26381	-0.51055
**H**	-3.03698	-4.74522	-0.02293
**H**	-0.14695	4.449166	-0.88064
**H**	4.99199	4.973684	0.595009

**H**	6.342212	4.058417	2.355608
**H**	5.197168	2.622242	2.551392
**H**	5.189734	-5.54873	-1.82567
**H**	5.867124	-3.95283	-0.16918
**H**	3.789569	-4.47136	-2.36281
**H**	-2.09792	-1.13232	2.326541
**H**	-6.26638	-0.44752	1.021544
**H**	-4.5747	0.805806	0.760913
**H**	-4.99175	2.489383	0.45934
**H**	-3.99576	-3.99571	1.984682
**H**	-2.31746	-3.45659	2.151342
**H**	1.770217	5.91654	-0.95088
**H**	1.401807	5.898051	0.774724
**H**	3.059262	6.136126	0.233196
**H**	6.963493	-1.99257	0.150882
**H**	6.572324	-0.57736	1.12858
**H**	6.823698	-0.4107	-0.60888

Table S3. XYZ-files of optimized geometry of H-Tyros ($FeC_{52}N_6H_{50}O_8Cl$) (B3LYP, GenECP, S =5/2). The basis sets: LANL2DZ for Fe atom; 6-31+G(d,p) for H atom**,** 6-31+G(d,p) for C, O, N and Cl atoms.

	**X**	**Y**	**Z**
**Fe**	-3.82921	-0.26085	-0.23024
**Cl**	-3.81708	-0.68702	-2.47847
**O**	3.073648	4.645119	-1.83367
**O**	3.782675	-0.3837	-0.93964
**O**	5.936113	-3.13429	-4.03864
**O**	5.847119	-3.7464	4.444821
**O**	6.555236	-1.00983	-3.83976
**O**	7.124175	2.82847	-1.97543
**O**	2.179435	5.479602	4.495764
**O**	5.843191	1.013954	-2. 748
**N**	-5.46977	0.918601	-0.132
**N**	-2.6936	1.407204	-0.22992
**N**	-2.19049	-1.32576	0.275485
**N**	-4.9628	-1.82571	0.367413
**N**	4.359476	-2.46495	-1.65545

**N**	3.693396	2.604767	-1.02439
**C**	-6.77284 0	0.516842	0.028652
**C**	-0.48022	0.411408	0.064514
**C**	-0.88635	-0.89903	0.243505
**C**	0.019166	-2.01152	0.431985
**C**	-0.75902	-3.12512	0.597351
**C**	-2.13193	-2.68252	0.495746
**C**	-3.22226	-3.52401	0.60678
**C**	-4.54459	-3.12358	0.534656
**C**	-5.68272	-4.00323	0.731436
**C**	-6.79658	-3.1978	0.733363
**C**	-6.33391	-1.85785	0.477217
**C**	-7.68136	1.63315	-0.15815
**C**	-7.17576	-0.76929	0.333329
**C**	-9.13421	1.533085	-0.06598
**C**	-9.97976	2.500419	0.31082
**C**	-7.32988	4.111614	-0.81268
**C**	-2.0905	5.052499	-0.91882
**C**	0.561429	3.293487	-0.34298
**C**	1.357987	2.971223	-1.63348
**C**	2.782129	3.500381	-1.52901
**C**	1.514455	-1.91506	0.357758
**C**	-6.89733	2.721568	-0.46664
**C**	2.026353	-2.0028	-1.10426
**C**	3.46637	-1.55011	-1.23039
**C**	-0.32675	-4.54157	0.822139
**C**	-5.66945	-5.45593	0.901636
**C**	-4.90035	-6.33304	0.246566
**C**	-8.22917	-3.60182	0.89417
**C**	-5.53115	2.260316	-0.43269
**C**	-4.43358	3.080417	-0.6334
**C**	-3.11624	2.686023	-0.51087
**C**	-1.98974	3.588812	-0.61849
**C**	-0.86921	2.840504	-0.38217
**C**	-1.32354	1.482737	-0.16032
**C**	5.791528	-2.22219	-1.83098

**C**	6.098063	-2.16304	-3.34489
**C**	6.635127	-3.34169	-1.17935
**C**	6.422097	-3.47075	0.31172
**C**	6.013415	-3.69452	3.088143
**C**	6.919957	-2.50657	1.198939
**C**	5.72316	-4.5519	0.851554
**C**	5.516293	-4.67079	2.225795
**C**	6.721723	-2.60821	2.569661
**C**	5.064401	2.980601	-0.72988
**C**	6.101871	2.302021	-1.63792
**C**	5.454338	2.71038	0.750357
**C**	4.578164	3.430849	1.750131
**C**	2.948037	4.77112	3.611187
**C**	4.60431	4.827457	1.862584
**C**	3.719629	2.724942	2.594261
**C**	2.908653	3.380867	3.520713
**C**	3.802282	5.496109	2.777687
**H**	0.599317	4.372692	-0.18046
**H**	0.581078	0.613557	0.0936 00
**H**	-3.03168	-4.56976	0.798368
**H**	-8.23491	-0.94574	0.457555
**H**	-2.56041	5.227739	-1.89171
**H**	-1.10586	5.520436	-0.94387
**H**	-2.68835	5.578243	-0.16747
**H**	-0.59874	-5.18439	-0.02161
**H**	0.754933	-4.60869	0.952264
**H**	-0.79214	-4.967	1.716222
**H**	1.069124	2.843397	0.516073
**H**	0.889517	3.462783	-2.48728
**H**	1.85815	-0.9723	0.788662
**H**	1.977455	-2.70981	0.9487
**H**	1.889563	-3.01617	-1.48986
**H**	1.43127	-1.33559	-1.73367
**H**	-4.62126	4.1202	-0.86251
**H**	4.013438	-3.36498	-1.95698
**H**	6.013994	-1.27627	-1.34434

**H**	7.68618	-3.12312	-1.39518
**H**	6.408649	-4.28321	-1.68618
**H**	7.485341	-1.66307	0.814582
**H**	5.343304	-5.32819	0.194865
**H**	4.977307	-5.52621	2.62264
**H**	7.115841	-1.86398	3.251035
**H**	5.376314	-4.55033	4.688411
**H**	6.510264	-0.27236	-3.19812
**H**	3.368453	1.683815	-0.759
**H**	5.14246	4.046439	-0.94237
**H**	6.499141	3.013505	0.869997
**H**	5.412704	1.633179	0.942225
**H**	5.261129	5.407047	1.222166
**H**	3.685007	1.641371	2.540261
**H**	2.252525	2.809386	4.170982
**H**	3.82602	6.575966	2.859525
**H**	1.634368	4.875698	5.01087
**H**	5.013345	0.644431	-1.61628
**H**	-9.56067	0.569215	-0.33238
**H**	-11.0489	2.324512	0.328534
**H**	-9.64214	3.478213	0.631534
**H**	-6.65448	4.573667	-1.53564
**H**	-8.33104	4.103633	-1.24821
**H**	-7.36191	4.761615	0.069239
**H**	1.35277	1.894537	-1.82374
**H**	-6.39401	-5.84867	1.612012
**H**	-4.98222	-7.39585	0.442261
**H**	-4.19869	-6.02836	-0.52122
**H**	-8.31291	-4.63928	1.220266
**H**	-8.77634	-3.51447	-0.05064
**H**	-8.74409	-2.97798	1.630438

Table S4. XYZ-files of optimized geometry of H-Styr ($FeC_{48}N_4H_{44}O_4Cl$) (B3LYP, GenECP, S = 5/2). The basis sets: LANL2DZ for Fe atom; 6-31+G(d,p) for H atom**,** 6-31+G(d,p) for C, O, N and Cl atoms.

	X	Y	Z

**Fe**	-0.12642	-0.31974	-0.24704
**Cl**	0.056247	-0.22986	2.05922
**O**	3.822949	-5.1322	2.148397
**O**	7.724691	0.053504	-0.66031
**O**	8.116765	1.618274	0.910306
**O**	5.297911	-6.30696	1.007244
**N**	-1.78555	-1.47264	-0.39142
**N**	0.991564	-1.96806	-0.56232
**N**	1.476221	0.807146	-0.72072
**N**	-1.2861	1.31761	-0.48027
**C**	-3.09036	-1.05032	-0.37757
**C**	3.188233	-0.93637	-0.8566
**C**	2.773883	0.382832	-0.86324
**C**	3.665914	1.51099	-1.02506
**C**	2.885682	2.633701	-0.97709
**C**	1.527063	2.179907	-0.77831
**C**	0.445975	3.029738	-0.64711
**C**	-0.86493	2.623621	-0.47442
**C**	-2.00178	3.524902	-0.38428
**C**	-3.12422	2.727814	-0.39934
**C**	-2.66038	1.366396	-0.43249
**C**	-3.99666	-2.18266	-0.29103
**C**	-3.50113	0.268439	-0.39795
**C**	-5.44483	-2.06948	-0.29551
**C**	-6.30905	-2.91174	0.306456
**C**	-3.62689	-4.74264	-0.20197
**C**	1.6004	-5.67354	-0.4497
**C**	4.238772	-3.83551	-0.85561
**C**	5.035184	-3.89897	0.483995
**C**	4.672403	-5.12756	1.299192
**C**	5.15663	1.412195	-1.16113
**C**	-3.20162	-3.30834	-0.24506
**C**	5.869378	1.339097	0.207161
**C**	7.31814	0.9211	0.077438
**C**	3.304103	4.067626	-1.08897
**C**	-1.97931	4.9778	-0.2915

**C**	-1.09287	5.714189	0.407084
**C**	-4.55922	3.148332	-0.32355
**C**	-1.83878	-2.84575	-0.30874
**C**	-0.73727	-3.68465	-0.32047
**C**	0.573183	-3.27372	-0.45476
**C**	1.69575	-4.18248	-0.54096
**C**	2.808351	-3.40482	-0.70554
**C**	2.354971	-2.02965	-0.71036
**C**	9.512162	1.256517	0.903364
**C**	6.440478	-6.37236	0.14487
**C**	-1.06539	7.17649	0.514145
**C**	-0.96847	9.976704	0.827139
**C**	-0.305	7.76767	1.537459
**C**	-1.76213	8.027039	-0.36289
**C**	-1.71769	9.406969	-0.20429
**C**	-0.2595	9.14968	1.69611
**C**	-7.77204	-2.82228	0.283545
**C**	-10.5889	-2.70155	0.344099
**C**	-8.48231	-2.0218	-0.62946
**C**	-8.5128	-3.57327	1.21231
**C**	-9.90279	-3.51196	1.246326
**C**	-9.87005	-1.95948	-0.59533
**H**	4.274477	-4.80908	-1.35217
**H**	4.245105	-1.12467	-0.97867
**H**	0.633328	4.090783	-0.71163
**H**	-4.56287	0.463232	-0.35664
**H**	1.364097	-5.98954	0.570443
**H**	2.543528	-6.14991	-0.72058
**H**	0.827195	-6.06881	-1.11451
**H**	3.111616	4.615647	-0.16105
**H**	4.370276	4.151948	-1.30409
**H**	2.763831	4.583259	-1.88837
**H**	4.763858	-3.14443	-1.51922
**H**	6.103982	-3.86784	0.265472
**H**	4.789435	-3.032	1.09635
**H**	5.433233	0.53149	-1.74419

**H**	5.538	2.27406	-1.71542
**H**	5.801649	2.286434	0.742852
**H**	5.38154	0.585298	0.834657
**H**	-0.91615	-4.74629	-0.23375
**H**	9.986017	1.920935	1.622061
**H**	9.936886	1.39733	-0.09121
**H**	9.636482	0.214945	1.202427
**H**	6.223861	-5.99792	-0.85822
**H**	6.693643	-7.4293	0.082747
**H**	7.288052	-5.82736	0.568307
**H**	-5.84943	-1.21108	-0.8231
**H**	-5.91104	-3.71906	0.912271
**H**	-2.92402	-5.38737	-0.73314
**H**	-3.69798	-5.11503	0.826194
**H**	-4.6084	-4.8665	-0.66422
**H**	-2.79473	5.48976	-0.79495
**H**	-0.34533	5.196013	1.26
**H**	-4.6567	4.233092	-0.37517
**H**	-5.01972	2.8256	0.615923
**H**	-5.14792	2.720555	-1.14072
**H**	0.245804	7.130668	2.221011
**H**	-2.32895	7.607705	-1.18526
**H**	-2.26079	10.04332	-0.89334
**H**	0.329464	9.580085	2.49749
**H**	-0.93299	11.05301	0.945386
**H**	-7.94736	-1.45815	-1.3841
**H**	-7.98661	-4.20393	1.920659
**H**	-10.4494	-4.09721	1.976408
**H**	-10.3962	-1.33804	-1.31066
**H**	-11.671	-2.65346	0.365174

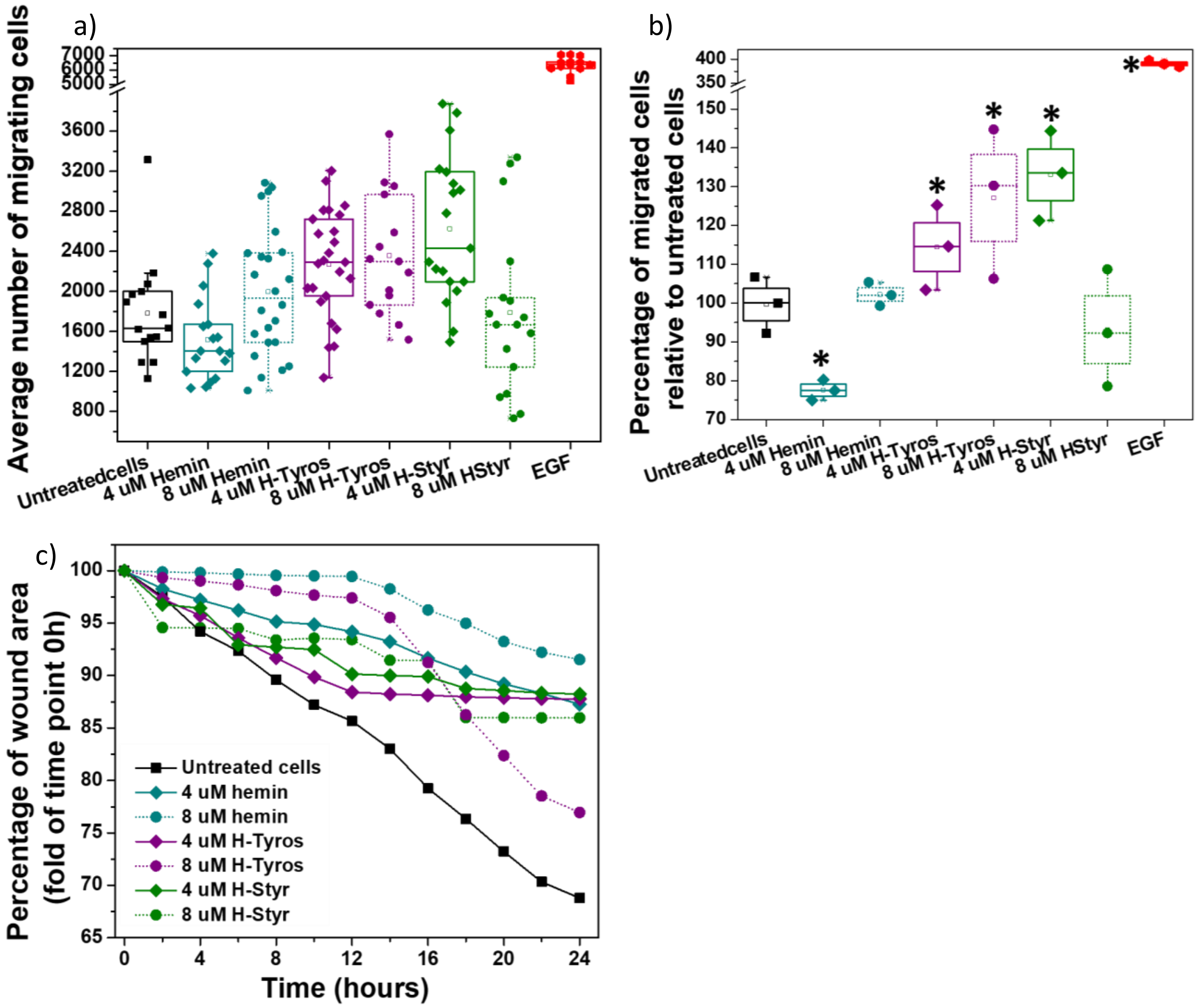

Figure S2. MDA-MB-231 cell migration in response to different compounds. a,b) The migration of cell through the transwell membranes towards the chemoattractant containing hemin, H-Tyros, or H-Styr, tested at the concentrations 4 and 8 µM. a) Box-whisker plot showing the distribution of the counted migrated cells. b) Percentage of migrated cells normalized to the count in the untreated cells (cells migrated towards the FBS-containing medium only). c) The effects of different compounds on cell migration, measured by scratch assay. Data are represented as mean ± S.D, n = 3. *, $P < 0.05$ versus the untreated cells left to migrate for 24 hours using a two-tailed unpaired student's t-test.

Video S1. The time-dependent migration of untreated MDA-MB-231 cells using a scratch assay, and the accompanying changes in the gap area. Scale bar: 800 µm.

Insert video 1.mp4 here

Video S2. The time-dependent migration of MDA-MB-231 cells, treated with 4 µM hemin, using a scratch assay, and the accompanying changes in the gap area. Scale bar: 800 µm.

Insert video 2.mp4 here

Video S3. The time-dependent migration of MDA-MB-231 cells, treated with 8 µM hemin, using a scratch assay, and the accompanying changes in the gap area. Scale bar: 800 µm.

Insert video 3.mp4 here

Video S4. The time-dependent migration of MDA-MB-231 cells, treated with 4 µM H-Styr, using a scratch assay, and the accompanying changes in the gap area. Scale bar: 800 µm.

Insert video 4.mp4 here

Video S5. The time-dependent migration of MDA-MB-231 cells, treated with 8 µM H-Styr, using a scratch assay, and the accompanying changes in the gap area. Scale bar: 800 µm.

Insert video 5.mp4 here

Video S6. The time-dependent migration of MDA-MB-231 cells, treated with 4 µM H-Tyros, using a scratch assay, and the accompanying changes in the gap area. Scale bar: 800 µm.

Insert video 6.mp4 here

Video S7. The time-dependent migration of MDA-MB-231 cells, treated with 8 µM H-Tyros, using a scratch assay, and the accompanying changes in the gap area. Scale bar: 800 µm.

Insert video 7.mp4 here

Intracellular ROS Generation: Influence of Radical Initiator Type and Culture Medium Components

The ROS detection study was initiated by testing the metabolic activity of MDA-MB-231 against different concentrations of tert-Butyl hydroperoxide (tBuOOH) and H_2O_2 as radical initiators for choosing a positive control with a cytocompatible concentration throughout the study. Although these compounds produce various types of radicals, they are collectively referred to as ROS (Reactive Oxygen Species). Within cells, these generated radicals typically lead to the oxidation of specific organelles, contributing to the induction of oxidative stress — a phenomenon associated with cancer and other diseases.[16,17] A decrease in the metabolic activity of MDA-MB-231

was observed with the increase in concentration of both tBuOOH and H_2O_2 **(Figure S2A)**. For the same concentration, tBuOOH showed more severe effects on cell viability than H_2O_2. This relates to the different sensitivity of MDA-MB-231 to the divergent radical species from these two radical generators, with a higher level of oxidative stress in the case of tBuOOH treatment.

Next, the optimum concentration of 2',7'–dichlorofluorescein diacetate (DCF-DA) was found by testing the ROS generation in 100 μM H_2O_2 and 10 μM tBuOOH-treated cells. The fluorescence intensity is mainly proportional to the amount of generated intracellular ROS, with the initial concentration of DCF-DA as a primary determinant factor for fluorescence generation. Here, the cell treatment with 10 μM DCF-DA did not induce significant changes in the ROS-mediated fluorescence reading due to tBuOOH nor H_2O_2 **(Figure S2B)**. Nevertheless, an enhancement in fluorescence due to 25 μM DCF-DA was detected with a higher level in H_2O_2- than in tBuOOH-treated cells. However, the fluorescence intensity increased significantly in 40 μM DCF-DA-treated cells, with higher levels of ROS generated after 24 hours than after 12 hours. The differences in fluorescence intensity due to the two ROS donors relate to their initial different concentrations and the reactivities of the resulting reactive species.

Based on the previous experiments, 25 μM DCF-DA was employed for evaluating the effects of hemin on the generation of intracellular ROS. **Figure S3A** shows the changes in DCF-fluorescence accompanying ROS generation in hemin-treated MDA-MB-231 cells. In general, the generation was proportional to hemin concentration, with significantly higher levels in the FBS-free medium than in the FBS-containing medium. These effects relate to the roles of hemin in the induction of $HO^{\bullet}$ radical generation upon its cellular uptake, as explained before.[18,19] This is accompanied by excessive generation of $HO^{\bullet}$ radicals, to be detected by DCF probe. However, this probe detects the generally generated ROS and is not sensitive to certain types of radicals. The lower fluorescence intensity in cells in FBS-containing media (**Figure S3B)** relates to the entrapment of hemin within the protein components of the medium and the lower cellular uptake in this medium compared to the FBS-free medium, as we illustrated previously.[20]

a)

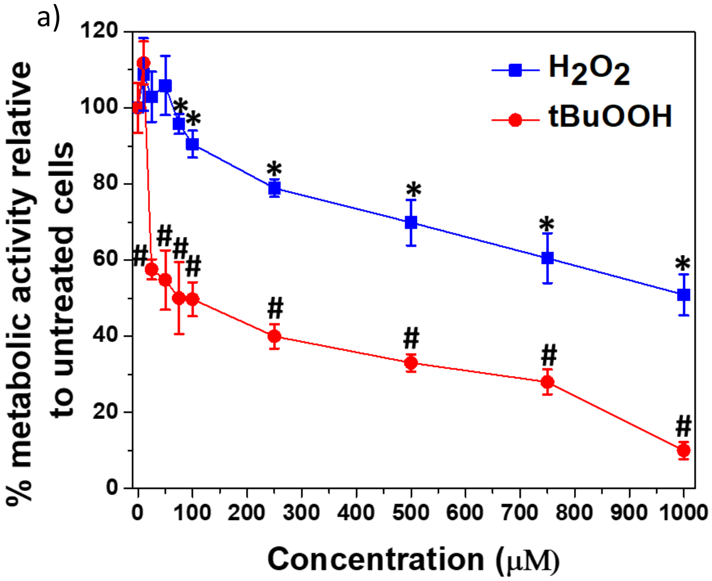

b)

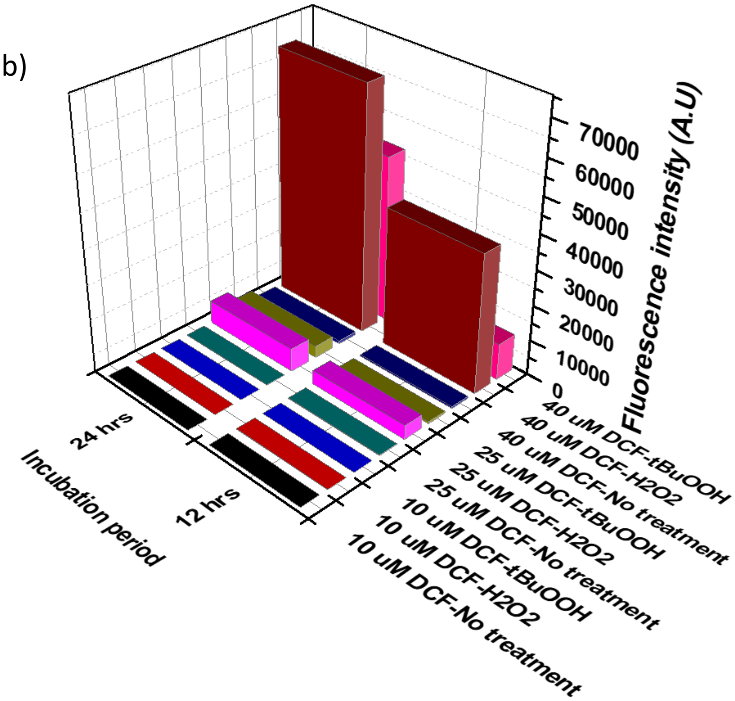

Figure S2. Effects of H_2O_2 and tBuOOH on the metabolic activity of MDA-MB-231 after 24 hours of culture (a) and the levels of ROS detected by DCF-fluorescence after 12 and 24 hours of culture (b). The cells were treated either directly with different concentrations of H_2O_2 and tBuOOH for assessment of cell viability, or with prior treatment with 10, 25 or 40 μM DCF-DA for monitoring the changes in intracellular ROS levels. Results are presented as mean ± S.D, n = 3. *, $P < 0.05$ versus the control group (untreated cells) in case of H_2O_2-treated cells; $^{\#}$, $P < 0.05$ versus the control group (untreated cells) in case of H_2O_2-treated cells using a two-tailed unpaired student t-test.

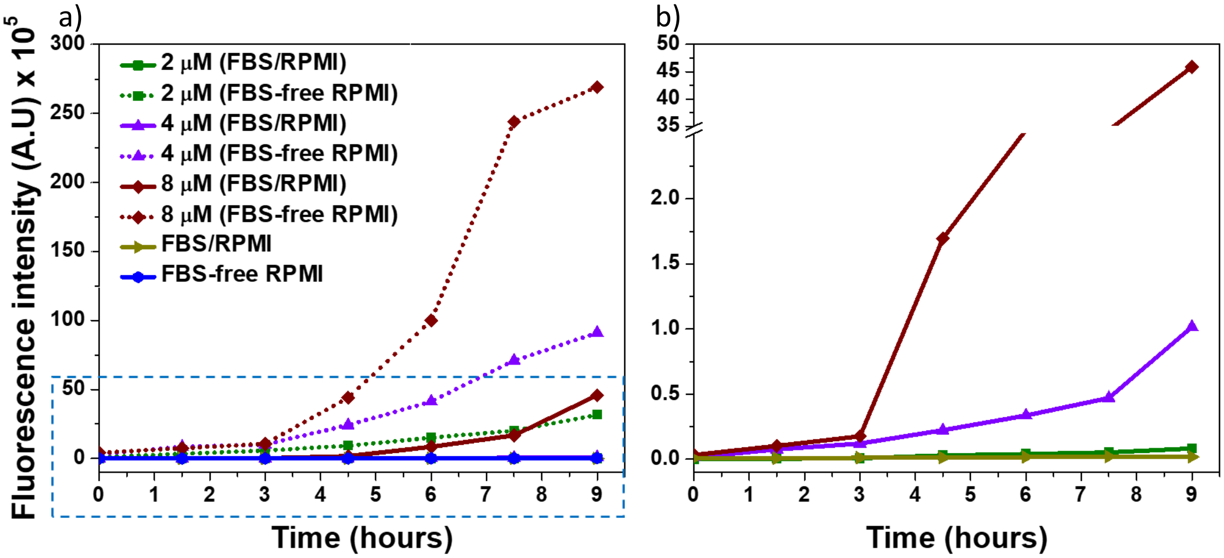

Figure S3. a) Kinetics of the changes in intracellular ROS levels revealed by the ROS-specific indicator DCF-DA and its fluorescence in MDA-MB-231 cells in response to 2, 4 and 8 µM hemin in FBS-free and FBS-containing media. b) The change in fluorescence in case of FBS-containing medium only with different treatments. The cells were treated with DCF-DA for 45 min, and then photographed after adding the different treatments using the real-time Incucyte® imaging system (phase contrast and green fluorescence signals). Results are presented as mean fluorescence intensity, n = 3.

Video S8. The time-dependent changes in levels of intracellular ROS, in DCF-DA-labelled MDA-MB-231 cells, without any treatment. Scale bar: 400 µm.

Insert video 8.mp4 here

Video S9. The time-dependent changes in levels of intracellular ROS, in unlabelled MDA-MB-231 cells, without any treatment. Scale bar: 400 µm.

Insert video 9.mp4 here

Video S10. The time-dependent changes in levels of intracellular ROS, in DCF-DA-labelled MDA-MB-231 cells, treated with 2 µM hemin. Scale bar: 400 µm.

Insert video 10.mp4 here

Video S11. The time-dependent changes in levels of intracellular ROS, in DCF-DA-labelled MDA-MB-231 cells, treated with 4 μM hemin. Scale bar: 400 μm.

Insert video 11.mp4 here

Video S12. The time-dependent changes in levels of intracellular ROS, in DCF-DA-labelled MDA-MB-231 cells, treated with 8 μM hemin. Scale bar: 400 μm.

Insert video 12.mp4 here

Video S13. The time-dependent changes in levels of intracellular ROS, in DCF-DA-labelled MDA-MB-231 cells, treated with 2 μM H-Tyros. Scale bar: 400 μm.

Insert video 13.mp4 here

Video S14. The time-dependent changes in levels of intracellular ROS, in DCF-DA-labelled MDA-MB-231 cells, treated with 4 μM H-Tyros. Scale bar: 400 μm.

Insert video 14.mp4 here

Video S15. The time-dependent changes in levels of intracellular ROS, in DCF-DA-labelled MDA-MB-231 cells, treated with 8 μM H-Tyros. Scale bar: 400 μm.

Insert video 15.mp4 here

Video S16. The time-dependent changes in levels of intracellular ROS, in DCF-DA-labelled MDA-MB-231 cells, treated with 2 μM H-Styr. Scale bar: 400 μm.

Insert video 16.mp4 here

Video S17. The time-dependent changes in levels of intracellular ROS, in DCF-DA-labelled MDA-MB-231 cells, treated with 4 μM H-Styr. Scale bar: 400 μm.

Insert video 17.mp4 here

Video S18. The time-dependent changes in levels of intracellular ROS, in DCF-DA-labelled MDA-MB-231 cells, treated with 8 μM H-Styr. Scale bar: 400 μm.

Insert video 18.mp4 here

The chemiluminescence signal and accompanied kinetics can distinguish between hemin and its derivatives

The different compounds were diluted in the phosphate buffer or FBS-free DMEM, followed by injection of the chemiluminescence (CL) reagents. In all solutions, hemin enhanced an initial flash kinetics luminescence, and the intensity, after reaching its transient amplitude, started to decrease gradually, reaching a steady level after around 30 min of reaction **(Figure S4A)**. The maximum intensity during that phase depended on the components of the testing solution. It reached 9.2×10^6 (20×10^6) and 3.8×10^6 (9.7×10^6) RLU in case of 4 μM (8 μM) hemin in phosphate buffer and FBS-free DMEM, respectively **(Figures S4A)**. However, the second flash kinetics, following the glow luminescence, started after 60 min of reaction with a low light yield, and the maximum intensity was the same in all solutions for the same hemin concentration. However, this amplitude was reached after 90 min of reactions in FBS-free DMEM, but after 120 min in the other solutions. A detailed explanation for how hemin can catalyze the H_2O_2/luminol-mediated CL reactions was reported before.[21,22]

H-Tyros showed different luminescence kinetics, where a sharp initial increase in luminescence intensity was observed, followed by a fast rate of decay. This one-phase of flash kinetics took place in all solutions and was followed by a long period of glow luminescence kinetics. Moreover, in contrast to hemin, the transient amplitudes were the highest in buffer, followed by DMEM **(Figures S3B)**. These results refer to the higher susceptibility of H-Tyros to the initial oxidation by H_2O_2 once mixed, catalysing of the CL reaction with generation of light. The phenol groups would be expected to act as electron-donating groups and prevent the oxidative effects of H_2O_2; however, these effects were significant with cancellation of the second flash luminescence only. Moreover, as the luminescence intensity following the initial decay was very low compared to that of hemin, the probability of glow-kinetics due to the released iron is low. These observations refer to the higher efficiency of H-Tyros to catalyse the CL reaction, with possible higher oxidizing power of the central iron, but becomes more resistant to H_2O_2-oxidation with lower catalytic efficiency overtime, compared to hemin.

Compared to other compounds, H-Styr showed the lowest efficiency to enhance the CL reactions, where a weak increase in the luminescence signal was detected once the CL reagents added to all tested solutions **(Figures S3C)**. This was followed by a slow rate of decay over the recording period, and this behaviour generally relates to the highest resistance of H-Styr to H_2O_2-oxidation.

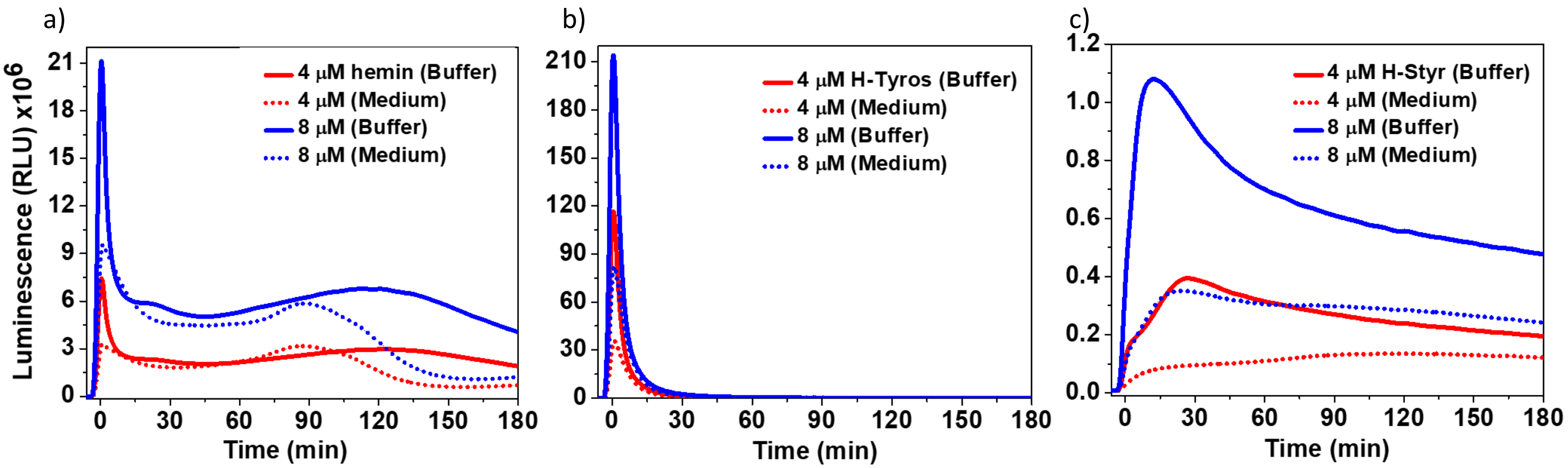

Figure S4. The luminescence kinetics following addition of 50 mM H_2O_2 and 1 mM luminol in phosphate buffer (50 mM, pH 7.4) to hemin-(a), H-Tyros-(b), and H-Styr (c)-containing phosphate buffer **(solid curves)** or FBS-free DMEM **(dotted curves)** at the concentrations 4 **(red curves)** and 8 μM **(blue curves).** Results are presented as mean luminescence intensity values, n = 3.

Binding free energy calculations.

Molecular Mechanics Poisson-Boltzmann Surface Area (MM-PBSA) method calculates the free energy (ΔG_{bind}) between a receptor and a ligand for every selected trajectory frame produced by the MD simulations as:

$$\Delta G_{bind} = G_{complex} - (G_{receptor} + G_{ligand}) \quad \text{(S1)}$$

where, $G_{complex}$, $G_{receptor}$, and G_{ligand} denote the absolute free energy of the complex, receptor (HOX-1), and ligands, respectively. The binding free energy can be described in terms of enthalpy and entropy contributions:

$$\Delta G_{bind} = \Delta H - T\Delta S \quad \text{(S2)}$$

where, $-T\Delta S$ is the conformational entropy contribution upon ligand binding. The enthalpy term (ΔH) is composed of the molecular mechanical energy in the gas phase ($\Delta E_{MM,gas}$), and the solvation free energy (ΔG_{solv}) and is given by the equation:

$$\Delta H = \Delta E_{MM,gas} + \Delta G_{solv} \quad \text{(S3)}$$

$\Delta E_{MM,gas}$ includes the internal energy change (ΔE_{int}), van der Waals (ΔE_{vdW}) and electrostatic energy (ΔE_{elec}) contributions:

$$\Delta E_{MM,gas} = \Delta E_{int} + \Delta E_{vdW} + \Delta E_{elec} \quad \text{(S4)}$$

Since the complex, receptor, and ligand structures are extracted from the same trajectory, the internal energy change (ΔE_{int}) is not considered. ΔG_{solv} consists of polar and non-polar energy contributions:

$$\Delta G_{solv} = \Delta G_{PB} + \Delta G_{NP} \quad \text{(S5)}$$

where, the polar solvation energy (ΔG_{PB}) is calculated using the nonpolar optimization method which separates the non-polar solvation interactions into the attractive (dispersion) and repulsive (cavity) terms (inp=1),[23] respectively. According to equation (S1), the enthalpy term (*ΔH*) of the free energy is now defined as the sum of the molecular mechanical energy in the gas phase predicted by the force field used, and the solvation free energy, which is calculated accordingly by each method. The electrostatic (ΔG_{PB}) energy is estimated by the Poisson−Boltzmann (PB) approach through a solvent-accessible surface area (*SASA*) term is used to express the hydrophobic contribution to solvation (ΔG_{NP}):

$$\Delta G_{NP} = \gamma * SASA + \beta \quad \text{(S6)}$$

Table S5. Binding affinities (in kcal mol^{-1}) of heme, hemin, H-Tyros, and H-Styr in the HOX-1 protein determined via molecular docking using the Autodock Vina software.

**Compound**	**Binding affinity**
Heme	-13.94
Hemin	-13.23
H-Tyros	-15.56
H-Styr	-10.71

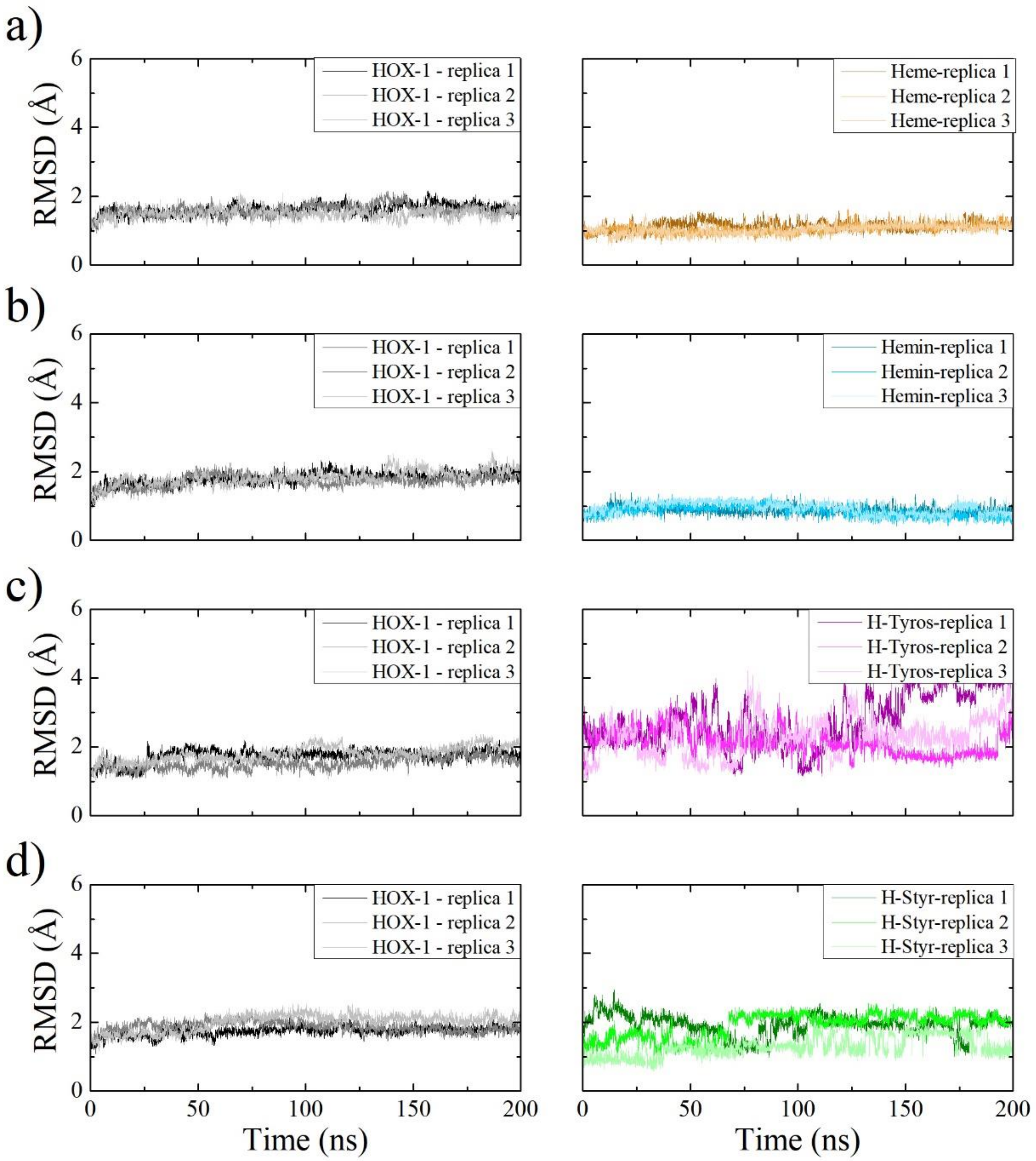

Figure S5. RMSD trajectory analyses of HOX-1 protein complexes with: a) heme, b) hemin, c) H-Tyros and d) H-Styr derivatives.

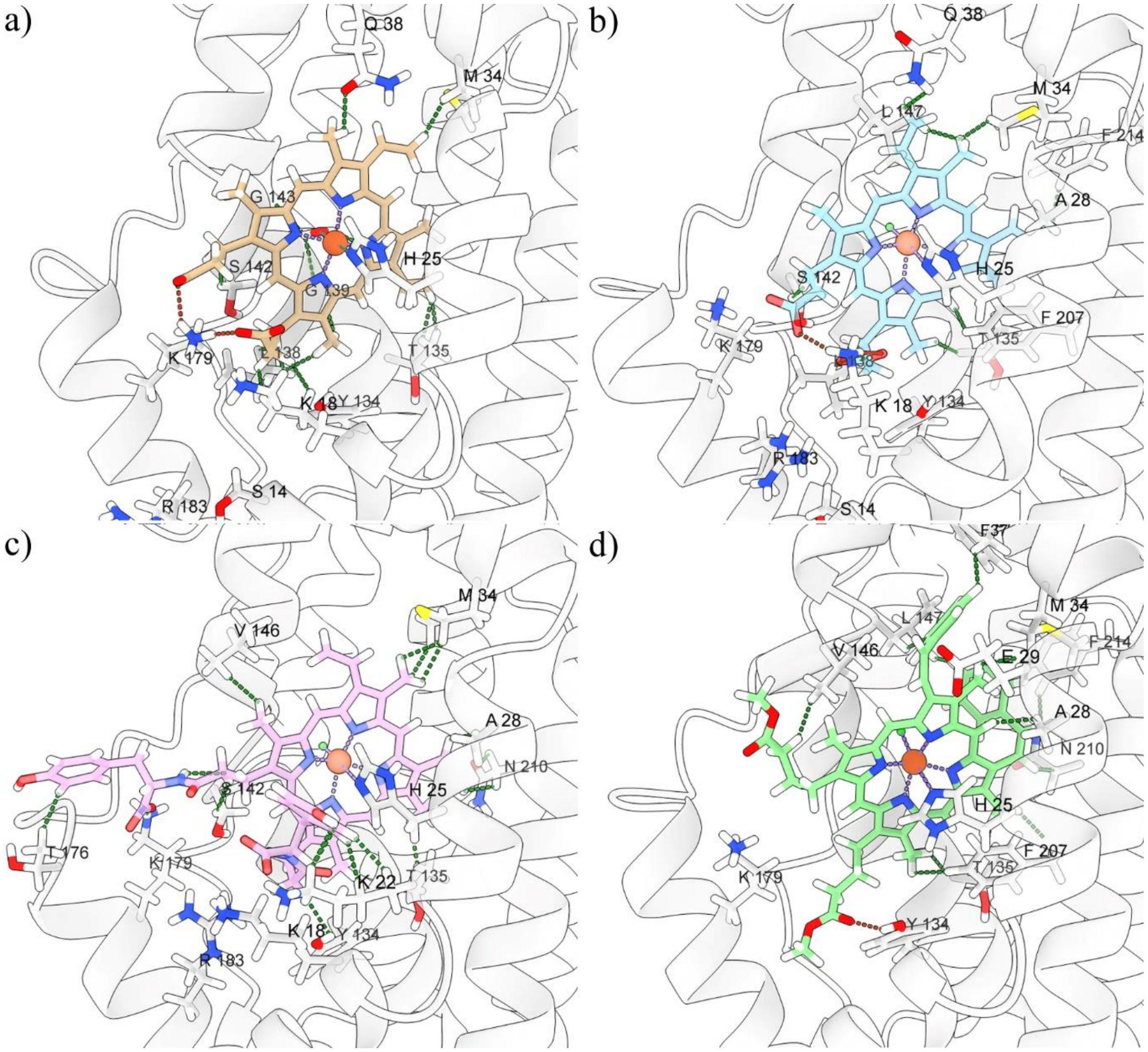

Figure S6. Centroid structure of: a) heme, b) hemin, c) H-Tyros and d) H-Styr derivatives in complex with HOX-1, determined from clustering conformational analysis of the MD simulation trajectories. Figure was prepared with ChimeraX.[24-26] Hydrogen bonds (red) and close contacts (green) with HOX-1 residues as also illustrated as dashed lines.

Table S6. Per-residue MM–PBSA energy decomposition of the HOX-1 complexes with heme, hemin, H-Tyros and H-Styr. Only the most favorable residues are shown. Uncertainties denote standard error of the mean (units in kcal mol^{-1}) and are included in parentheses.

Residue	van der Waals		Electrostatic		Polar Solvation		Total energy	
**Heme**								
**K^{18}**	-0.66	(0.01)	-61.79	(0.19)	58.31	(0.16)	-4.14	(0.03)
**K^{22}**	-0.11	(0.00)	-17.27	(0.05)	16.95	(0.05)	-0.43	(0.00)
**H^{25}**	-1.60	(0.01)	-7.24	(0.03)	5.85	(0.03)	-2.99	(0.01)
**M^{34}**	-0.81	(0.00)	0.09	(0.00)	0.20	(0.01)	-0.52	(0.01)
**Y^{134}**	-1.18	(0.01)	-5.28	(0.05)	6.17	(0.03)	-0.29	(0.02)
**L^{138}**	-1.23	(0.00)	0.65	(0.01)	-0.75	(0.01)	-1.34	(0.01)
**G^{139}**	-1.84	(0.00)	1.14	(0.01)	-0.29	(0.01)	-0.99	(0.01)
**D^{140}**	-0.49	(0.00)	7.70	(0.01)	-7.73	(0.02)	-0.51	(0.02)
**G^{143}**	-1.45	(0.00)	-0.27	(0.01)	0.36	(0.01)	-1.36	(0.01)
**V^{146}**	-0.83	(0.00)	-0.32	(0.00)	0.60	(0.00)	-0.55	(0.00)
**L^{147}**	-1.27	(0.01)	-0.20	(0.00)	0.35	(0.00)	-1.12	(0.01)
**K^{179}**	-0.15	(0.01)	-56.46	(0.17)	53.72	(0.15)	-2.90	(0.04)
**R^{183}**	0.08	(0.01)	-36.87	(0.12)	32.98	(0.10)	-3.80	(0.02)
**F^{207}**	-2.27	(0.01)	-0.83	(0.00)	1.48	(0.00)	-1.62	(0.01)
**Hemin**								
**K^{18}**	-0.66	(0.01)	-76.44	(0.19)	72.38	(0.16)	-4.72	(0.03)
**K^{22}**	-0.13	(0.00)	-28.72	(0.07)	28.13	(0.07)	-0.72	(0.00)
**A^{28}**	-1.10	(0.00)	-1.04	(0.00)	1.23	(0.01)	-0.91	(0.01)
**M^{34}**	-0.82	(0.00)	0.02	(0.00)	0.26	(0.00)	-0.54	(0.00)
**R^{85}**	-0.02	(0.00)	-20.03	(0.01)	19.46	(0.01)	-0.59	(0.00)
**R^{136}**	-0.58	(0.00)	-24.06	(0.01)	23.03	(0.02)	-1.61	(0.01)
**L^{138}**	-1.57	(0.00)	1.24	(0.01)	-1.23	(0.01)	-1.56	(0.01)
**G^{139}**	-1.83	(0.00)	2.02	(0.01)	-1.06	(0.01)	-0.87	(0.01)
**S^{142}**	-1.99	(0.01)	-0.08	(0.03)	1.00	(0.03)	-1.08	(0.01)
**G^{143}**	-1.82	(0.01)	-2.11	(0.01)	2.05	(0.01)	-1.89	(0.01)
**V^{146}**	-1.04	(0.00)	-1.92	(0.00)	1.88	(0.00)	-1.07	(0.00)
**L^{147}**	-1.82	(0.00)	-1.69	(0.00)	1.55	(0.00)	-1.96	(0.01)
**K^{179}**	-0.13	(0.01)	-72.10	(0.22)	69.50	(0.19)	-2.73	(0.04)
**R^{183}**	-0.05	(0.01)	-52.52	(0.16)	48.23	(0.12)	-4.34	(0.05)
**F^{207}**	-2.43	(0.01)	-1.10	(0.00)	2.08	(0.01)	-1.45	(0.01)
**I^{211}**	-0.50	(0.00)	-0.29	(0.00)	0.23	(0.00)	-0.56	(0.00)
**H-Tyros**								
**K^{18}**	-1.62	(0.01)	-55.65	(0.12)	54.97	(0.11)	-2.30	(0.02)

**K^{22}**	-1.00	(0.01)	-35.37	(0.15)	34.76	(0.14)	-1.60	(0.02)
**A^{28}**	-1.09	(0.00)	-0.98	(0.00)	1.27	(0.01)	-0.80	(0.01)
**M^{34}**	-0.89	(0.00)	0.03	(0.00)	0.29	(0.00)	-0.58	(0.00)
**R^{136}**	-0.56	(0.00)	-22.10	(0.02)	21.31	(0.03)	-1.35	(0.01)
**L^{138}**	-1.62	(0.01)	1.60	(0.01)	-1.05	(0.01)	-1.07	(0.01)
**G^{139}**	-2.01	(0.00)	1.48	(0.01)	-0.88	(0.01)	-1.41	(0.01)
**S^{142}**	-2.62	(0.01)	-2.17	(0.04)	4.21	(0.04)	-0.58	(0.01)
**G^{143}**	-2.12	(0.01)	-2.20	(0.01)	2.32	(0.01)	-2.00	(0.01)
**G^{144}**	-0.55	(0.00)	-0.95	(0.00)	0.86	(0.00)	-0.63	(0.00)
**Q^{145}**	-0.61	(0.01)	-2.55	(0.02)	2.55	(0.02)	-0.61	(0.01)
**V^{146}**	-1.40	(0.01)	-1.69	(0.00)	1.63	(0.00)	-1.46	(0.01)
**L^{147}**	-1.79	(0.01)	-1.46	(0.00)	1.25	(0.00)	-2.00	(0.01)
**K^{148}**	-0.06	(0.00)	-20.88	(0.02)	20.38	(0.02)	-0.57	(0.00)
**T^{176}**	-0.52	(0.01)	0.24	(0.03)	0.13	(0.03)	-0.16	(0.01)
**K^{179}**	-0.38	(0.01)	-66.84	(0.14)	65.15	(0.12)	-2.07	(0.04)
**F^{207}**	-2.56	(0.01)	-0.93	(0.00)	1.90	(0.01)	-1.58	(0.01)
**F^{214}**	-0.59	(0.00)	-0.36	(0.00)	0.59	(0.00)	-0.36	(0.00)
**H-Styr**								
**A^{28}**	-1.47	(0.00)	0.11	(0.00)	0.42	(0.01)	-0.94	(0.01)
**M^{34}**	-2.06	(0.01)	-0.09	(0.00)	0.88	(0.01)	-1.26	(0.01)
**L^{138}**	-1.82	(0.01)	0.50	(0.00)	-0.14	(0.01)	-1.46	(0.01)
**S^{142}**	-3.02	(0.01)	-1.79	(0.01)	3.41	(0.02)	-1.40	(0.02)
**G^{143}**	-0.64	(0.00)	-0.17	(0.00)	0.10	(0.00)	-0.72	(0.00)
**Q^{145}**	-1.19	(0.01)	-0.64	(0.01)	1.16	(0.01)	-0.66	(0.01)
**V^{146}**	-2.87	(0.01)	-0.72	(0.00)	0.86	(0.00)	-2.73	(0.01)
**L^{147}**	-1.54	(0.00)	-0.56	(0.00)	0.75	(0.00)	-1.35	(0.01)
**I^{150}**	-1.04	(0.00)	-0.24	(0.00)	0.38	(0.00)	-0.90	(0.00)
**F^{207}**	-2.67	(0.01)	-0.51	(0.00)	1.42	(0.01)	-1.76	(0.01)
**I^{211}**	-0.80	(0.00)	0.14	(0.00)	-0.13	(0.00)	-0.78	(0.00)
**F^{214}**	-1.25	(0.00)	0.05	(0.00)	0.61	(0.00)	-0.59	(0.00)

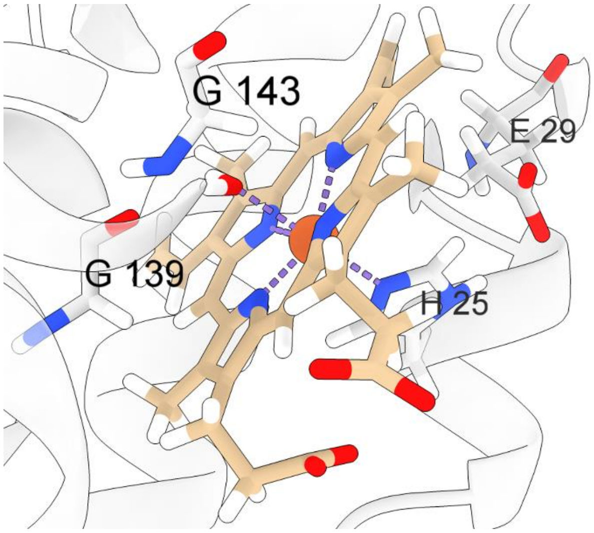

Figure S7. The centroid structure of the HOX-1 protein complex with heme, showcasing the water-mediated hydrogen bonding interaction involving the Fe(III) coordinated water and G^{139} and G^{143} residues. Residues H^{25} and E^{29}, which are hydrogen bonded for the majority of the simulation time, are also illustrated.

Table S7. Average center of mass distances (in Å) between the Fe(III) coordinated water molecule of heme and the chloride atom of hemin, H-Tyros, and H-Styr derivatives with the A^{140}, G^{143}, and L^{147} HOX-1 residues, calculated from three replicate trajectories. Uncertainties are indicated in parentheses.

		**Derivative**		
**Distance**	**Heme**	**Hemin**	**H-Tyros**	**H-Styr**
A^{140}-H_2O	5.5 (0.6)	–	–	–
G^{143}-H_2O	4.4 (0.5)	–	–	–
L^{147}-H_2O	6.9 (0.3)	–	–	–
A^{140}-Cl	–	5.6 (0.5)	5.7 (0.4)	7.0 (0.3)
G^{143}-Cl	–	4.1 (0.3)	4.0 (0.2)	4.9 (0.3)
L^{147}-Cl	–	6.2 (0.4)	6.2 (0.3)	6.4 (0.4)

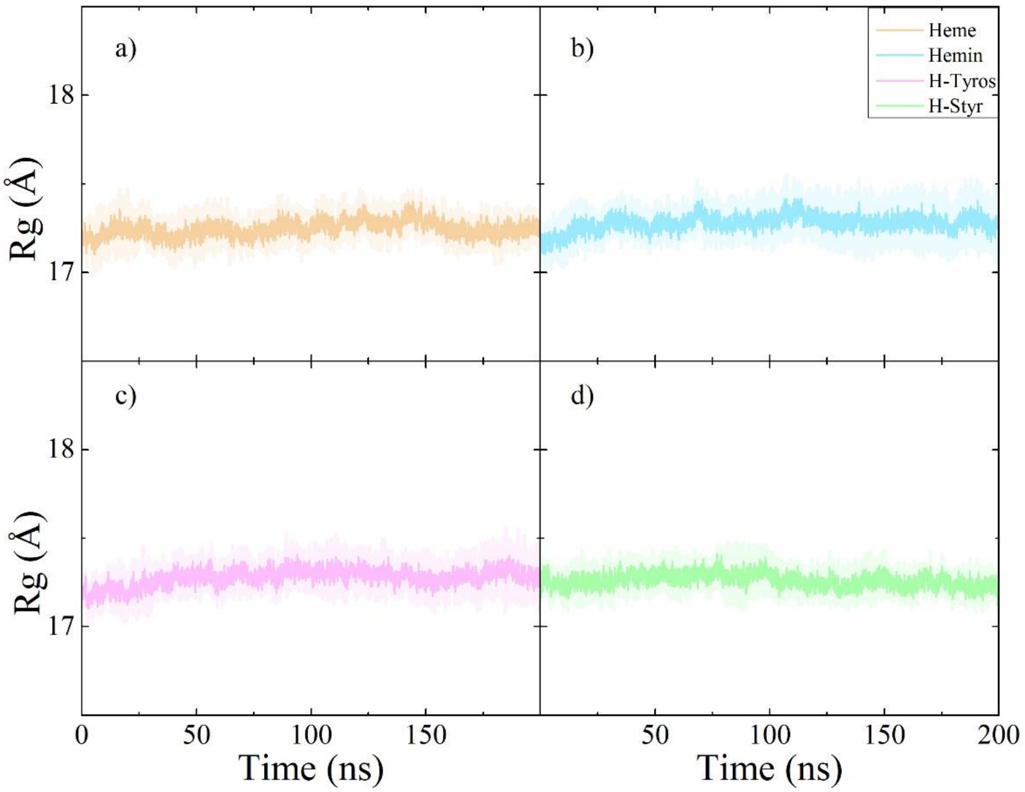

Figure S8. HOX-1 protein's radius of gyration (Rg) in complex with **(a)** heme, **(b)** hemin, **(c)** H-Tyros, and **(d)** H-Styr derivatives. Solid lines depict the average Rg value, while shaded regions indicate the respective standard deviation from three independent simulations. Average values are equal to **(a)** 17.25 ± 0.07, **(b)** 17.28 ± 0.09, **(c)** 17.28 ± 0.08 and **(d)** 17.26 ± 0.07 Å, respectively.

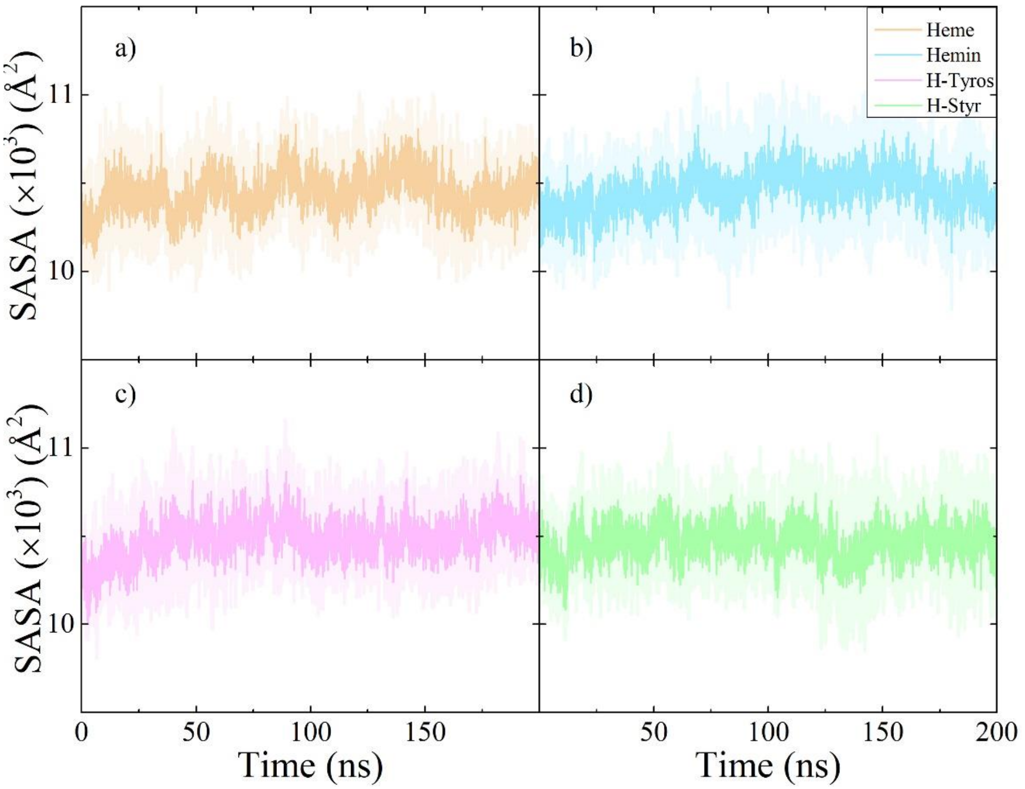

Figure S9. The solvent-accessible surface area (SASA) of the HOX-1 protein in complex with: a) heme, b) hemin, c) H-Tyros, and d) H-Styr derivatives. Solid lines indicate the average SASA value, while shaded regions represent the respective standard deviation from three independent simulations. Average values are equal to a) 10.44 ± 0.18, b) 10.46 ± 0.21, c) 10.48 ± 0.19 and d) 10.46 ± 0.20 $\times 10^3$Å^2, respectively.

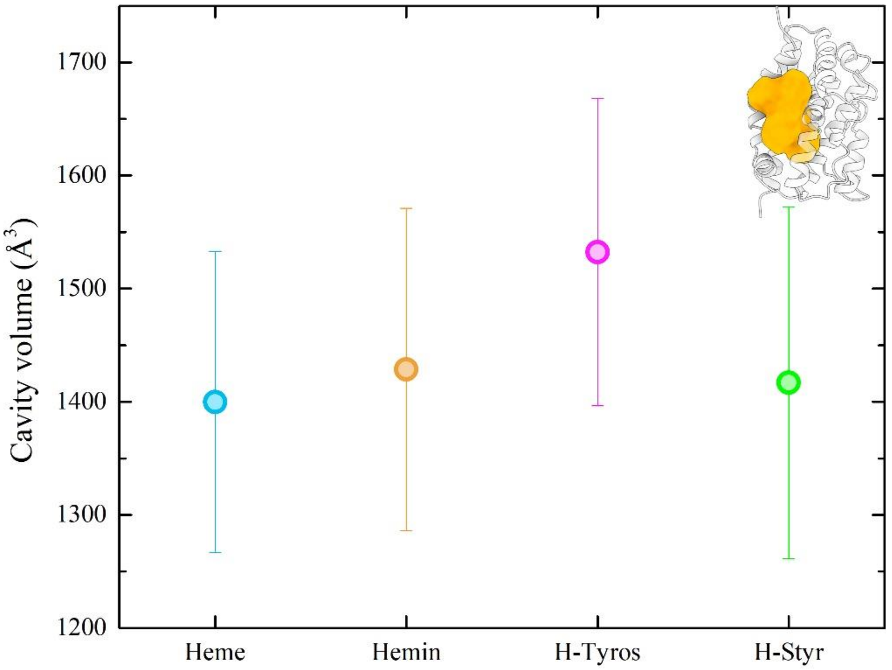

Figure S10. Calculated average pocket volume of the HOX-1 protein including the standard deviation in its complexes with heme, hemin, H-Tyros, and H-Styr derivatives, measured over the last 100 ns of all trajectories using the MDpocket program.[27] Average values are equal to 1399.9 ± 133.1, 1428.5 ± 142.4, 1532.5 ± 135.9 and 1416.8 ± 155.5 Å^3, respectively.

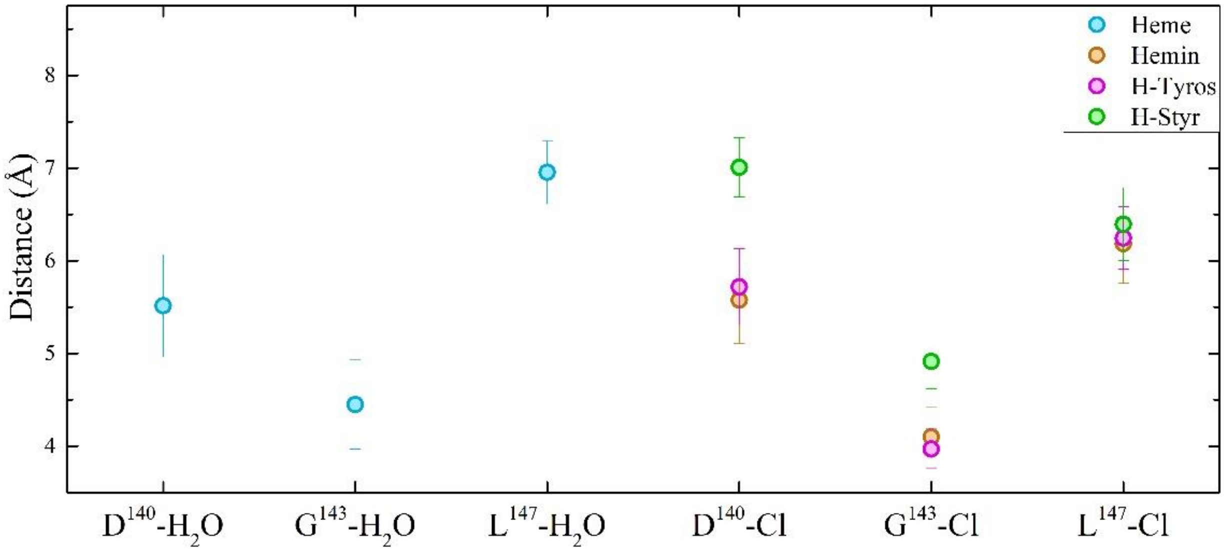

Figure S11. The average COM distances between the Fe(III) coordinated water and chlorine atom of heme and hemin, respectively, and D^{140}, G^{143} and L^{147} residues in the heme, hemin, H-Tyros, and H-Styr complexes with HOX-1.

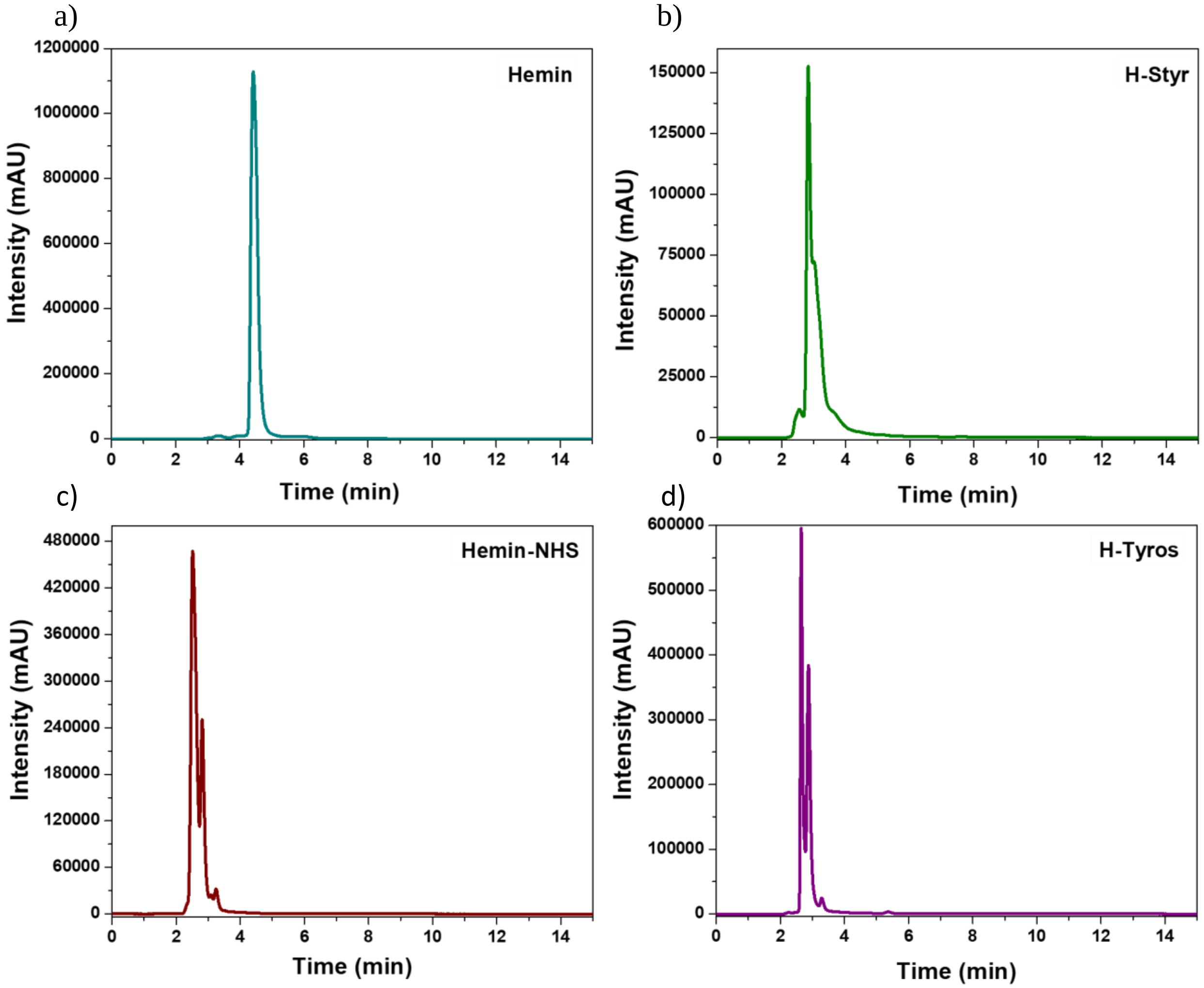

Figure S12. Comparison of the HPLC chromatograms of: a) hemin, b) H-styr, c) hemin-N-oxysuccinimide ester (hemin-NHS) conjugate and d) H-Tyros.

Table S8. Grid box sizes used during the molecular docking calculations.

Compound	Grid box edge dimension (Å)
**Heme**	56.7
**Hemin**	56.7
**H-Tyros**	69.3
**H-Styr**	72.4

References

(1) Lad, L.; Schuller, D. J.; Shimizu, H.; Friedman, J.; Li, H.; Ortiz de Montellano, P. R.; Poulos, T. L. Comparison of the Heme-Free and -Bound Crystal Structures of Human Heme Oxygenase-1. *J. Biol. Chem.* **2003**, *278* (10), 7834–7843. https://doi.org/10.1074/jbc.M211450200.

(2) Eastman, P.; Swails, J.; Chodera, J. D.; McGibbon, R. T.; Zhao, Y.; Beauchamp, K. A.; Wang, L.-P.; Simmonett, A. C.; Harrigan, M. P.; Stern, C. D.; Wiewiora, R. P.; Brooks, B. R.; Pande, V. S. OpenMM 7: Rapid Development of High Performance Algorithms for Molecular Dynamics. *PLOS Comput. Biol.* **2017**, *13* (7), e1005659. https://doi.org/10.1371/journal.pcbi.1005659.

(3) Case DA, Darden TA, Cheatham TEI, Simmerling CL, Wang J, Duke RE, Luo R, Walker RC, Zhang W, Merz KM, Roberts B, Hayik S, Roitberg A, Seabra G, Swails J, Goetz AW, Kolossváry I, Wong KF, Paesani F, Vanicek J, Wolf RM, Liu J, Wu X, Brozell SR, Steinbrecher, K. A. AMBER12. **2012**.

(4) Stylianaki, E.-A.; Mouchlis, V. D.; Magkrioti, C.; Papavasileiou, K. D.; Afantitis, A.; Matralis, A. N.; Aidinis, V. Identification of Two Novel Chemical Classes of Autotaxin (ATX) Inhibitors Using Enalos Asclepios KNIME Nodes. *Bioorg. Med. Chem. Lett.* **2024**, *103*, 129690. https://doi.org/10.1016/j.bmcl.2024.129690.

(5) Papadopoulou, D.; Drakopoulos, A.; Lagarias, P.; Melagraki, G.; Kollias, G.; Afantitis, A. In Silico Identification and Evaluation of Natural Products as Potential Tumor Necrosis Factor Function Inhibitors Using Advanced Enalos Asclepios KNIME Nodes. *Int. J. Mol. Sci.* **2021**, *22* (19), 10220. https://doi.org/10.3390/ijms221910220.

(6) Vanquelef, E.; Simon, S.; Marquant, G.; Garcia, E.; Klimerak, G.; Delepine, J. C.; Cieplak, P.; Dupradeau, F.-Y. R.E.D. Server: A Web Service for Deriving RESP and ESP Charges and Building Force Field Libraries for New Molecules and Molecular Fragments. *Nucleic Acids Res.* **2011**, *39*, W511–W517. https://doi.org/10.1093/nar/gkr288.

(7) Dupradeau, F.-Y.; Pigache, A.; Zaffran, T.; Savineau, C.; Lelong, R.; Grivel, N.; Lelong, D.; Rosanski, W.; Cieplak, P. The R.E.D. Tools: Advances in RESP and ESP Charge Derivation and Force Field Library Building. *Phys. Chem. Chem. Phys.* **2010**, *12* (28), 7821. https://doi.org/10.1039/c0cp00111b.

(8) Bayly, C. I.; Cieplak, P.; Cornell, W.; Kollman, P. A. A Well-Behaved Electrostatic Potential Based Method Using Charge Restraints for Deriving Atomic Charges: The RESP Model. *J. Phys. Chem.* **1993**, *97* (40), 10269–10280. https://doi.org/10.1021/j100142a004.

(9) Wang, F.; Becker, J. -P.; Cieplak, P.; Dupradeau, F. R.E.D. Python: Object Oriented Programming for Amber Force Fields. *47th Am. Chem. Soc. Natl. Meet.* **2014**, 296.

(10) Becke, A. D. Density-Functional Thermochemistry. III. The Role of Exact Exchange. *J. Chem. Phys.* **1993**, *98* (7), 5648–5652. https://doi.org/10.1063/1.464913.

(11) Stephens, P. J.; Devlin, F. J.; Chabalowski, C. F.; Frisch, M. J. Ab Initio Calculation of Vibrational Absorption and Circular Dichroism Spectra Using Density Functional Force Fields. *J. Phys. Chem.* **1994**, *98* (45), 11623–11627. https://doi.org/10.1021/j100096a001.

(12) Hehre, W. J.; Ditchfield, R.; Pople, J. A. Self—Consistent Molecular Orbital Methods. XII. Further Extensions of Gaussian—Type Basis Sets for Use in Molecular Orbital Studies of Organic Molecules. *J. Chem. Phys.* **1972**, *56* (5), 2257–2261. https://doi.org/10.1063/1.1677527.

(13) Wang, J.; Cieplak, P.; Kollman, P. A. How Well Does a Restrained Electrostatic Potential (RESP) Model Perform in Calculating Conformational Energies of Organic and Biological Molecules? *J. Comput. Chem.* **2000**, *21* (12), 1049. https://doi.org/10.1002/1096-987X(200009)21:12<1049::AID-JCC3>3.3.CO;2-6.

(14) Seminario, J. M. Calculation of Intramolecular Force Fields from Second- derivative Tensors. *Int. J. Quantum Chem.* **1996**, *60* (7), 1271–1277. https://doi.org/10.1002/(SICI)1097-461X(1996)60:7<1271::AID-QUA8>3.3.CO;2-J.

(15) Zheng, S.; Tang, Q.; He, J.; Du, S.; Xu, S.; Wang, C.; Xu, Y.; Lin, F. VFFDT: A New Software for Preparing AMBER Force Field Parameters for Metal-Containing Molecular Systems. *J. Chem. Inf. Model.* **2016**, *56* (4), 811–818. https://doi.org/10.1021/acs.jcim.5b00687.

(16) Cerutti, P. A. Prooxidant States and Tumor Promotion. *Science (80-.).* **1985**, *227* (4685), 375–381. https://doi.org/10.1126/science.2981433.

(17) Stadtman, E. R.; Berlett, B. S. Reactive Oxygen-Mediated Protein Oxidation in Aging and Disease. *Drug Metab. Rev.* **1998**, *30* (2), 225–243. https://doi.org/10.3109/03602539808996310.

(18) Bhattacharyya, A.; Chattopadhyay, R.; Mitra, S.; Crowe, S. E. Oxidative Stress: An Essential Factor in the Pathogenesis of Gastrointestinal Mucosal Diseases. *Physiol. Rev.* **2014**, *94* (2), 329–354. https://doi.org/10.1152/physrev.00040.2012.

(19) Kord Forooshani, P.; Pinnaratip, R.; Polega, E.; Tyo, A. G.; Pearson, E.; Liu, B.; Folayan, T. O.; Pan, L.; Rajachar, R. M.; Heldt, C. L.; Lee, B. P. Hydroxyl Radical Generation through the Fenton-like Reaction of Hematin- And Catechol-Functionalized Microgels. *Chem. Mater.* **2020**, *32* (19), 8182–8194. https://doi.org/10.1021/acs.chemmater.0c01551.

(20) Alsharabasy, A. M.; Glynn, S.; Farràs, P.; Pandit, A. Protein Nitration Induced by Hemin/NO: A Complementary Mechanism through the Catalytic Functions of Hemin and NO-Scavenging. *Nitric Oxide* **2022**, *124*, 49–67. https://doi.org/10.1016/j.niox.2022.04.005.

(21) Yi, Z.; Ren, Y.; Li, Y.; Chen, B.; Li, Y.; Long, F.; Zhu, A. Highly Intensive and Long-Lasting Chemiluminescence “Three-in-One” Hemin/Fe3O4@MoS2 Hybrid Nanozyme Powered Lab-on-Fiber Device for Ochratoxin A Immunoassay. *Sensors Actuators B Chem.* **2023**, *392*, 134056. https://doi.org/10.1016/j.snb.2023.134056.

(22) Alsharabasy, A. M.; Farràs, P.; Pandit, A. Hemin as a Molecular Probe for Nitric Oxide Detection in Physiological Solutions: Experimental and Theoretical Assessment. *Anal. Chem.* **2024**, *96* (19), 7763–7771. https://doi.org/10.1021/acs.analchem.4c01516.

(23) Tan, C.; Tan, Y.-H.; Luo, R. Implicit Nonpolar Solvent Models. *J. Phys. Chem. B* **2007**, *111* (42), 12263–12274. https://doi.org/10.1021/jp073399n.

(24) Meng, E. C.; Goddard, T. D.; Pettersen, E. F.; Couch, G. S.; Pearson, Z. J.; Morris, J. H.; Ferrin, T. E. UCSF ChimeraX: Tools for Structure Building and Analysis. *Protein Sci.* **2023**, *32* (11), e4792. https://doi.org/10.1002/pro.4792.

(25) Pettersen, E. F.; Goddard, T. D.; Huang, C. C.; Meng, E. C.; Couch, G. S.; Croll, T. I.; Morris, J. H.; Ferrin, T. E. UCSF ChimeraX: Structure Visualization for Researchers, Educators, and Developers. *Protein Sci.* **2021**, *30* (1), 70–82. https://doi.org/10.1002/pro.3943.

(26) Goddard, T. D.; Huang, C. C.; Meng, E. C.; Pettersen, E. F.; Couch, G. S.; Morris, J. H.; Ferrin, T. E. UCSF ChimeraX: Meeting Modern Challenges in Visualization and Analysis. *Protein Sci.* **2018**, *27* (1), 14–25. https://doi.org/10.1002/pro.3235.

(27) Schmidtke, P.; Bidon-Chanal, A.; Luque, F. J.; Barril, X. MDpocket: Open-Source Cavity Detection and Characterization on Molecular Dynamics Trajectories. *Bioinformatics* **2011**, *27* (23), 3276–3285. https://doi.org/10.1093/bioinformatics/btr550.
